# Tetraimidazolium macrocycle: a versatile building block and precursor for box-type coordination cages

**DOI:** 10.1039/d5ra05896a

**Published:** 2025-10-02

**Authors:** Fang Wang, Kai Hua

**Affiliations:** a School of Chemistry and Chemical Engineering, Yan'an University Yan'an 716000 P. R. China wangf@yau.edu.cn; b Center of Basic Molecular Science (CBMS), Department of Chemistry, Tsinghua University Beijing 100084 China

## Abstract

This study describes a facile synthesis of the imidazolium-based cyclophane H_4_-1(PF_6_)_4_ from 1-(1*H*-imidazol-1-ylmethyl)-1*H*-imidazole and 1,4-bis(bromomethyl)benzene, followed by anion exchange. Slipped π–π stacking interactions between the flexible cyclophane and aromatic sulfonate anions—including the 1,5-naphthalenedisulfonate dianion (1,5-nds), 2,6-naphthalenedisulfonate dianion (2,6-nds), and 2,7-naphthalenedisulfonate dianion (2,7-nds)—induce one-dimensional (1D) self-assembly, forming nanochannels. Significantly, a metallocage, Ag_4_(1)_2_(PF_6_)_4_ is derived from the corresponding tetraimidazolium salt *via* reaction with Ag_2_O and subsequent transmetalation. This cyclophane-based metallocage possesses an optimal size for binding acetonitrile and exhibits excellent selectivity for acetonitrile encapsulation, mediated by weak N⋯Ag interactions.

## Abstract 2

Facile tetraimidazolium macrocycle synthesis enables π-stacking-driven self-assembly and selective acetonitrile binding in a derived metallocage.

## Graphical abstract

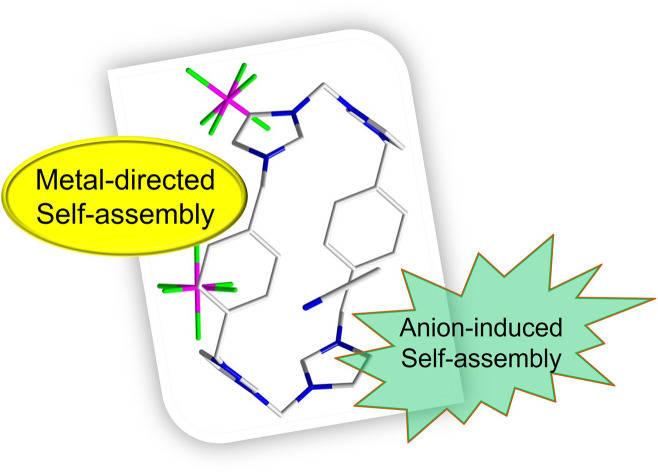

## Introduction

The design and synthesis of well-defined artificial macrocycles capable of sophisticated molecular recognition remain pivotal objectives in supramolecular chemistry, driven by applications in targeted binding, delivery, and separation.^1^ Such macrocycles serve as indispensable supramolecular building blocks, enabling the construction of complex architectures with tunable physicochemical properties.^2^ Among these, cyclic polyimidazolium salts—particularly those featuring four *N*-heterocyclic carbene (NHC) units—have garnered significant recent attention.^3^ These macrocycles function as versatile receptors for anions or π-electron-rich guests,^4^ and critically, as precursors for cyclic poly-NHC ligands and their metal complexes.^5^

While notable purely organic macrocycles (*e.g.*, “blue box”,^6^ “Texas-sized box”,^7^ “naphthocage”^8^) exhibit selective, stimulus-responsive guest encapsulation,^9^ metallocages incorporating NHC ligands and coinage metals (Cu, Ag, Au) offer structurally tunable cavities with enhanced functionality.^10–12^ For instance, Pöthig *et al.* demonstrated calix[4]imidazolium[2]pyrazolate-based pillarplexes encapsulating linear guests.^13^ Furthermore, poly-NHCs enable rapid access to diverse supramolecular assemblies *via* metal–carbene bonds.^14^ Nevertheless, significant gaps persist: (1) the self-assembly behavior of cyclic polyimidazolium salts, particularly anion-dependent configurations in the solid state, remains underexplored;^15^ (2) examples of discrete “box-type” metallocages, especially with silver(i) ions exclusively in bridging positions between two macrocyclic ligands, are scarce due to challenges associated with macrocycle size and flexibility;^16^ and (3) the potential of these systems for cavity-specific engineering towards targeted separation, distinct from stimulus-responsive capture/release, is largely untapped.

To address these challenges, rational design of specific cyclic tetrakis-imidazolium salt structures is crucial. We optimized the preparation of a novel tetra-nitrogen heterocyclic tetra-anionic precursor compound, H_4_-1(PF_6_)_4_. This method affords high yield, eliminates the need for column chromatography, and enables gram-scale synthesis.^17^ We employed H_4_-1(PF_6_)_4_ to investigate anion-directed self-assembly in the solid state and, notably, for the synthesis of coinage metal coordination boxes. Specifically, we describe the preparation and structural characterization of discrete Ag(i) and Au(i) metallocages in which four metal ions are bridged between two cyclic tetra-NHC ligands through M–C carbene bonds, forming a well-defined cavity. This work harnesses inherent metallocage design principles—in contrast to folding-directed assembly in conventional macrocycles—enabling structurally precise complexes with potential for tailored functionality.

## Results and discussion

### Synthesis and characterization of macrocyclic tetraimidazolium salt

**Scheme 1:**
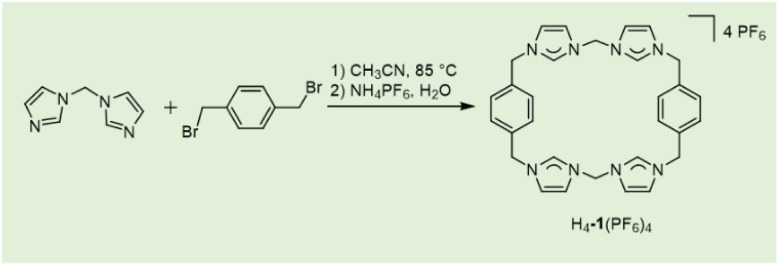
Synthesis of the macrocyclic tetraimidazolium salt H_4_-1(PF_6_)_4_.

Macrocyclic *tetra*-imidazolium salt, H_4_-1(PF_6_)_4_, synthesized *via* a two-step procedure (Scheme 1) with an overall yield of 76%. The synthesis commenced with a 1 : 1 mixture of 1-(1*H*-imidazol-1-ylmethyl)-1*H*-imidazole and 1,4-bis(bromomethyl)benzene in acetonitrile. Following a 48-hour reflux and subsequent anion exchange with NH_4_PF_6_, the target compound H_4_-1(PF_6_)_4_ was obtained by re-crystallization from water/acetonitrile. This compound exhibits high solubility in polar organic solvents such as acetonitrile, *N*,*N*-dimethylformamide, and dimethyl sulfoxide.

**Fig. 1:**
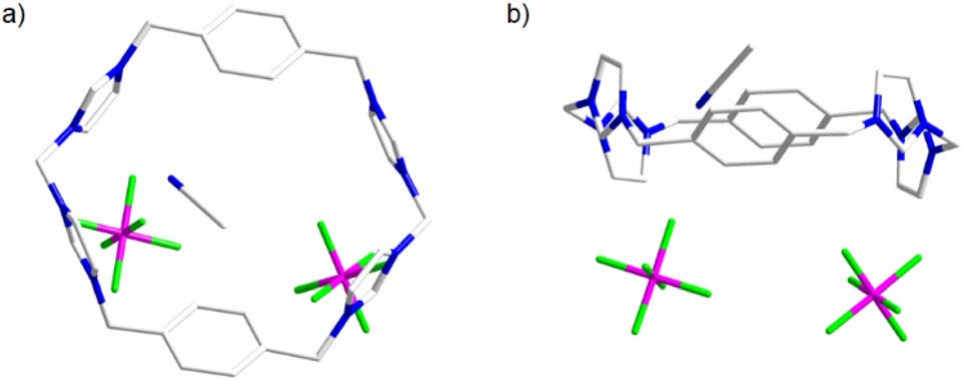
(a) Top view and (b) side view showing the sticks form of H_4_-1(PF_6_)_4_·2CH_3_CN·2H_2_O. The hydrogen atom and H_2_O molecules are omitted for clarity. The anions (PF_6_^−^) are located outside the macrocyclic.

The macrocycle H_4_-1(PF_6_)_4_ is characterized by ^1^H and ^13^C{^1^H} NMR spectroscopy in DMSO-*d*_6_. A single resonance at *δ* = 9.49 ppm (^1^H NMR) and *δ* = 137.9 ppm (^13^C NMR) was observed for the four equivalent NCHN protons and NCN carbon atoms, respectively. Besides, the macrocyclic structure was further confirmed by high-resolution electrospray ionization (HR-ESI) mass spectrometry, where the most intense peaks in positive ion mode *m*/*z* = 216.4185 (calcd for [H_4_-1(PF_6_)]^3 +^ 216.4125) and *m*/*z* = 397.1032 (calcd for [H_4_-1(PF_6_)_2_]^2 +^ 397.1011) exhibited isotopic distributions matching theoretical simulations(Fig. S1 and S2, SI). Single crystals of H_4_-1(PF_6_)_4_·2CH_3_CN·2H_2_O suitable for X-ray diffraction were grown by slow evaporation of an acetonitrile/water solution. As shown in Fig. 1, the NCHN groups of the imidazolium rings adopt an outward orientation, contrasting with literature reports where such groups point toward the macrocycle center.^18^ This tetracationic macrocycle features multiple aromatic π-surfaces (benzene rings), a large flexible cavity, and structural adaptability. Notably, CH_3_CN may act as a templating agent during cyclization, as evidenced by its occupancy within the cavity of H_4_-1(PF_6_)_4_ in the crystal structure.

### Supramolecular assembly *via* external anion binding and cooperative interactions

**Fig. 2:**
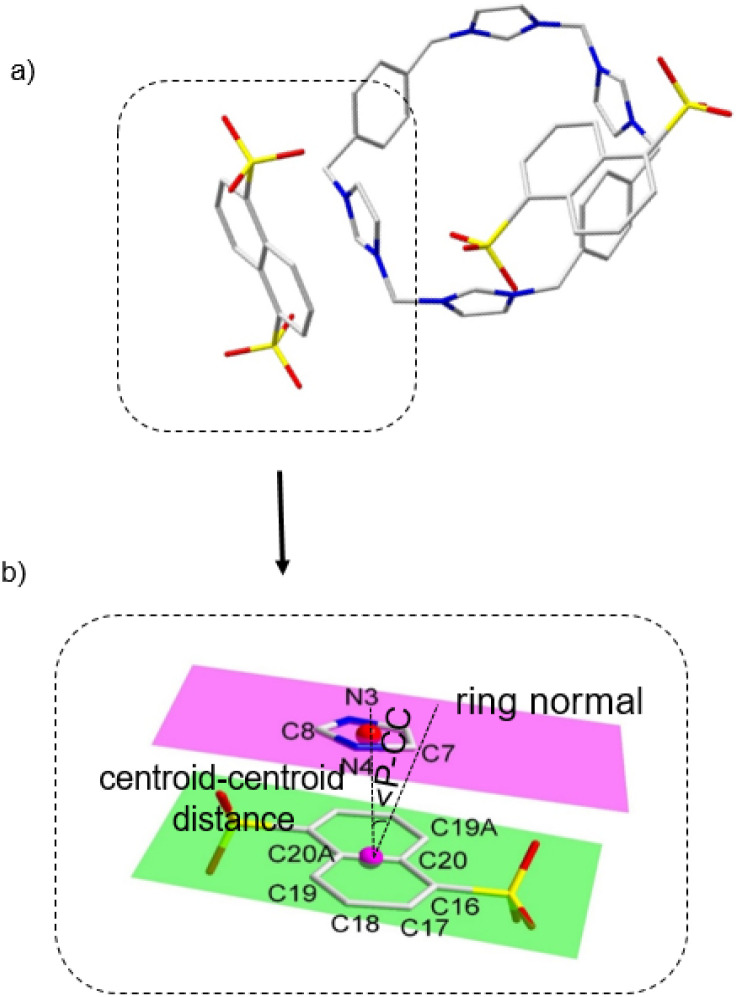
(a) The binding mode between (H_4_-1) and 1,5-naphthalene disulfonate dianion (1,5-nds) in stick form. (b) Expanded π–π donor–acceptor interaction stacking part in stick form. All the other molecules and atoms have been omitted for clarity. The possible π–π interactions were inferred from the following selected.

To elucidate the influence of guest molecules on supramolecular architecture, we performed anion exchange on the macrocyclic complex H_4_-1(PF_6_)_4_ with 1,5-naphthalene disulfonate (Na_2_1,5-nds). Single-crystal analysis of [(H_4_-1)·(1,5-nds)_2_·2CH_3_CN·9H_2_O] revealed an unexpected exo-binding mode: the dianion (1,5-nds) occupies the ‘chair face’ of a neighboring macrocycle, stabilized by π–π interactions (3.627 Å centroid distance; 18.06° displacement angle), C–H⋯O hydrogen bondinginteratomic distances [Å]: C(8)⋯C(20A) 3.727(4), N(4)⋯C(19) 3.744(0), C(7)⋯C(20) 3.728(4), C(7)⋯C(16) 3.854(4);and C–H⋯π interactions: [2.683(2) Å] (SI data). Critically, these cooperative forces drive the assembly into 1D solvent-filled channels (Fig. 2b), enhancing structural stability through synergy (Table S1, Fig. S8 and S9, SI).

Prompted by this exclusive exo-binding behavior, we systematically investigated aromatic sulfonate isomerism. Analysis of [(H_4_-1)(2,7-nds)_2_·2CH_3_CN·5H_2_O] confirmed the generality of external binding, with 2,7-nds residing entirely outside the cavity. Notably, while π-contacts weakened (>3.5 Å O-aromatic distances), shorter C–H⋯O bonds (<3.0 Å) emerged – a trend replicated in the 2,6-isomer complex (Table S1, SI). This consistent preference for exo-binding across isomers demonstrates that self-assembly is directed by complementary anion-π interactions and hydrogen bonding (Fig. S10 and S11, SI). Additionally, further UV-vis studies revealed no apparent charge-transfer bands in the long-wavelength region for complexes (H_4_-1)(nds)_2_, compared to (H_4_-1)(PF_6_)_4_ (Fig. S12, SI).

**Fig. 3:**
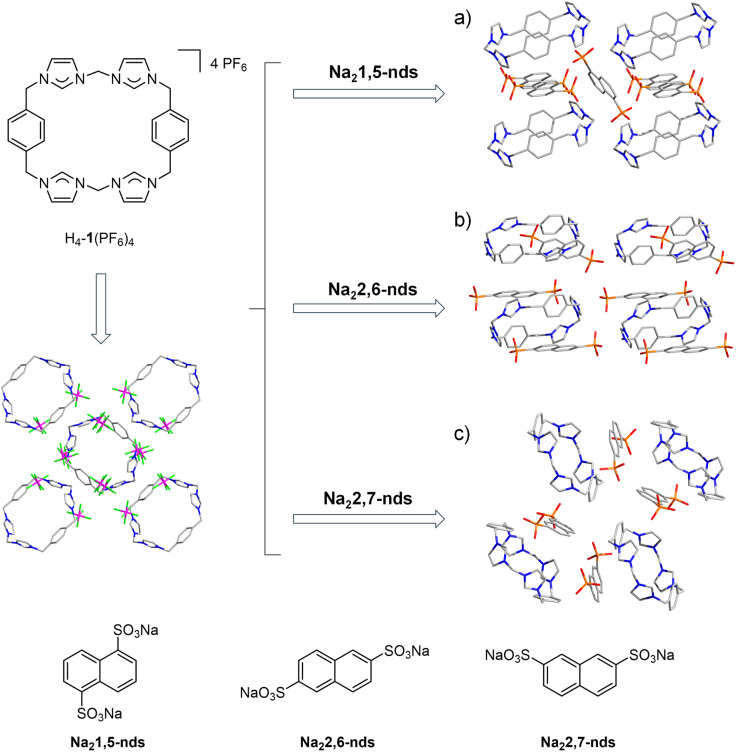
Anion exchange in the macrocyclic complex H_4_-1(PF_6_)_4_ with naphthalene disulfonate (Na_2_nds) and crystal packing diagrams of (a) [H_4_-1(1,5-nds)_2_], (b) H_4_-1(2,6-nds)_1_._5_ and (c) [H_4_-1(2,7-nds)_2_] (hydrogen atoms, CH_3_CN and H_2_O molecules omitted for clarity).

Structural comparisons further show that H_4_-1^4+^ adapts its conformation (folding/extension) to optimize anion binding. The steric bulk of sulfonates prevents cavity threading, explaining the universal formation of discrete exo-complexes (distinct from pseudorotaxanes). Thus, anion geometry fundamentally dictates the supramolecular architecture, with π-stacking and H-bonding acting as cooperative pillars of stability (Fig. 3).

### Box-type silver(i) and gold(i) tetracarbene assemblies with solvent-adaptive cavities

**Scheme 2:**
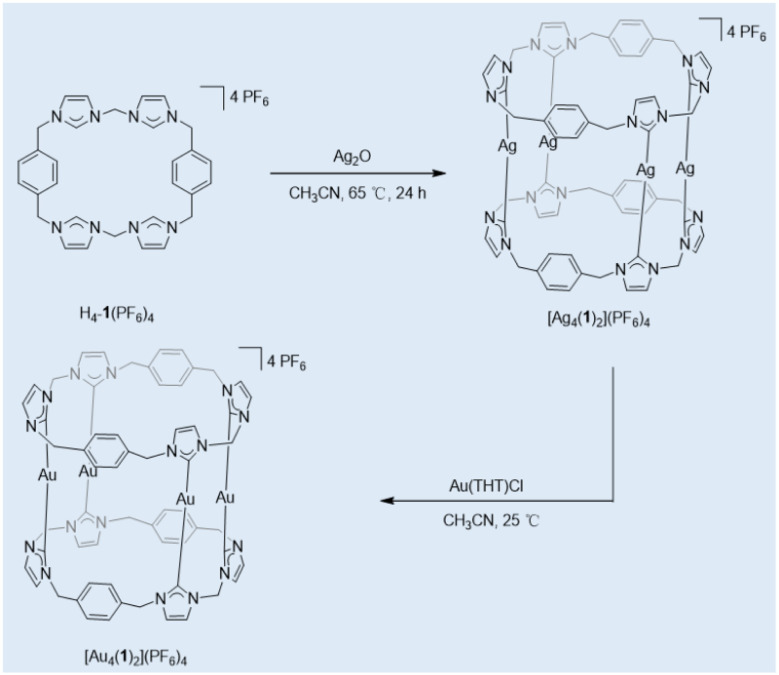
Preparation of tetranuclear silver(i) octacarbene complexes and their transmetalation reactions to tetranuclear gold(i) octacarbene complexes.

Building upon the structural insights gained from the anion-directed assemblies, we explored the metal coordination chemistry of the tetraimidazolium macrocycle H_4_-1(PF_6_)_4_, utilizing it as an NHC precursor. Treatment of H_4_-1(PF_6_)_4_ with 2.5 equivalents of Ag_2_O yielded the tetranuclear silver(i) complex Ag_4_(1)_2_(PF_6_)_4_ as an off-white solid (Scheme 2). Comprehensive characterization by ^1^H and ^13^C{^1^H} NMR spectroscopy and HR-ESI mass spectrometry confirmed the structure (Fig. S3 and S4, SI) The disappearance of the characteristic NCHN proton resonances of H_4_-1(PF_6_)_4_ and the appearance of a single singlet at *δ* = 181.7 ppm in the ^13^C{^1^H} NMR spectrum, assigned to the carbene carbon atoms, are diagnostic of successful NHC–Ag bond formation, consistent with literature values for related complexes.^19^ HR-ESI-MS (positive ions) further corroborated the tetranuclear formulation, displaying the highest intense peak at *m*/*z* = 861.0232, corresponding to the dication [Ag_4_(1)_2_(PF_6_)_2_]^2+^ (calcd 861.0176) (Fig. S6, SI).

**Fig. 4:**
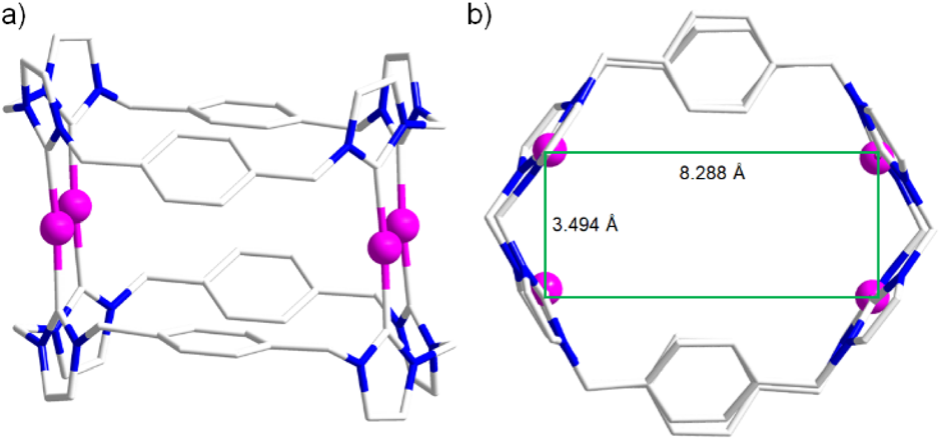
Side view (a) and top view (b) of molecular structure of box-type complex cation [Ag_4_(1)_2_]^4+^ (hydrogen atoms omitted for clarity). Selected bond distances (Å) and bond angles (°): range Ag⋯Ag, 3.494–8.288; Ag–C_NHC_ bond lengths, 2.082(3)–2.091(3); C_NHC_–Ag–C_NHC_ bond angles, 167.22(11)–170.05(11).

To obtain structural insights, anion exchange of Ag_4_(1)_2_(PF_6_)_4_ with NaBPh_4_ afforded Ag_4_(1)_2_(BPh_4_)_4_, from which single crystals suitable for X-ray diffraction were grown by slow diffusion of diethyl ether into an acetonitrile solution (Fig. 4). The structure unequivocally revealed the expected box-type complex cation [Ag_4_(1)_2_]^4+^ (solvated with 10 CH_3_CN molecules). Four silver(i) ions are sandwiched between two *tetra*-NHC ligands derived from H_4_-1^4+^. The observed metrical parameters [Ag–C_NHC_ bond lengths, 2.082(3)–2.091(3) Å; C_NHC_–Ag–C_NHC_ bond angles, 167.22(11)–170.05(11) °] fall within the typical range for related silver(i) polycarbene assemblies.^20^ Consistent with the box-type structure defined by the two *tetra*-NHC ligands, the four Ag(i) ions adopt an essentially planar rectangular arrangement as shown in Fig. 4b.

**Fig. 5:**
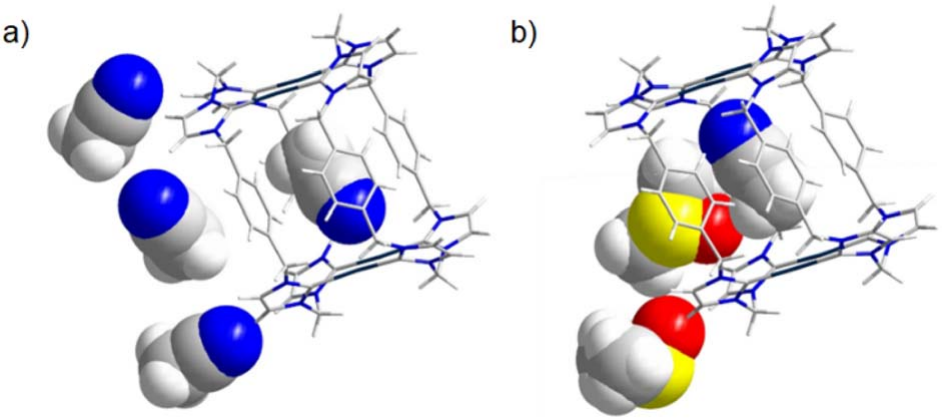
Orientation of the acetonitrile molecule (space-filling model) trapped inside the hollow cavity of box-like complex cation [Ag_4_(1)_2_]^4+^: (a) obtained through diethyl ether diffusion into pure acetonitrile; (b) obtained through diethyl ether diffusion into DMSO/acetonitrile. Tetraphenylborate anions are omitted for clarity.

Remarkably, identical crystallization of Ag_4_(1)_2_(BPh_4_)_4_ from acetonitrile/dimethylsulfoxide instead yielded crystals with an acetonitrile guest tightly encapsulated in the previously vacant cavity (Fig. 5), wherein the CH_3_CN nitrogen atom coordinates weakly to two Ag(i) centers (Ag⋯N = 3.128 Å and 3.037 Å, respectively). Although dimethylsulfoxide (DMSO) is present in the crystallization medium, it is excluded from the cavity and remains entirely external to the host structure. This observation clearly demonstrates selective molecular recognition modulated by solvent environment. To gain deeper insight into this phenomenon, we employed the VOIDOO program to calculate the cavity volume of the cage. The computed volume was found to be 84 Å^3^ (Fig. S15, SI). Compared to similar cage structures reported in the literature,^21*a*,*b*^ the cavity size of our cage exhibits better compatibility with the molecular volume of acetonitrile (52 Å^3^, as calculated by DFT at the B3LYP/6-31G* level; Table S5, SI; ref. 21*c*). Furthermore, computational studies at the B3LYP/6-31G*/LANL2DZ level successfully quantified the weak encapsulation interaction with acetonitrile, yielding a binding energy of −0.582 kcal mol^−1^ (Tables S3–S5, SI). Both architectures expose catalytically relevant Ag(i) sites, with the empty cavity structure offering an accessible reaction space and the host–guest variant exhibiting supramolecular trapping capability. Such adaptive behavior positions this system as a promising candidate for potential multifunctional applications spanning from catalysis to molecular sensing.^21*d*^

Having established the structural and functional properties of the silver-NHC assembly, we exploited its utility as a carbene transfer precursor to access the corresponding gold complex—motivated by the known efficacy of silver(i)–NHCs as transmetalation agents.^22^ Specifically, stirring an acetonitrile solution of Ag_4_(1)_2_(PF_6_)_4_ with 4 equiv of [AuCl(THT)] at ambient temperature for 24 h afforded the tetranuclear gold(i) complex Au_4_(1)_2_(PF_6_)_4_ as a beige solid in 63% yield (Scheme 2). Comprehensive characterization confirmed complete transmetalation: ^1^H NMR spectroscopy revealed the disappearance of silver-carbene signatures, while HR-ESI mass spectrometry exhibited two diagnostic peaks at *m*/*z* = 447.0971 (calcd for [Au_4_(1)_2_]^4+^: 447.0878) and 644.4461 (calcd for [Au_4_(1)_2_(PF_6_)]^3+^: 644.4387), unambiguously verifying the tetranuclear gold framework (Fig. S5 and S7, SI).

## Conclusions

In summary, we have synthesized and characterized the macrocyclic tetraimidazolium salt H_4_-1(PF_6_)_4_ as a versatile platform for supramolecular assembly and coordination chemistry. Single-crystal analyses demonstrate that the geometry of aromatic disulfonate anions (nds) fundamentally dictates the resulting supramolecular architecture through cooperative non-covalent interactions—primarily slipped π–π stacking and C–H⋯O hydrogen bonding—leading to the exclusive formation of discrete exo-complexes and 1D nanochannels. The anion-dependent self-assembly underscores the power of these interactions in directing complex structures. Furthermore, H_4_-1(PF_6_)_4_ acts as an effective NHC precursor, enabling the synthesis of the tetranuclear metallocage Ag_4_(1)_2_(PF_6_)_4_. This metallocage features an adaptable cavity exhibiting remarkable selectivity for encapsulating acetonitrile *via* weak N⋯Ag interactions. Transmetalation successfully afforded the analogous gold(i) complex. The demonstrated anion-directed assembly control and the metallocage's selective guest binding highlight the potential of this macrocyclic scaffold. Future efforts will focus on designing larger cyclic poly-NHC ligands derived from this platform to create tailored cavities for enhanced recognition of ions and small molecules.

## Conflicts of interest

There are no conflicts to declare.

## Author contributions

All authors conceptualized the research. The manuscript was written through contributions of all authors. All authors have given approval to the final version of the manuscript.

## Supplementary Material

RA-015-D5RA05896A-s001

RA-015-D5RA05896A-s002

## Data Availability

CCDC 2468088–2468094 contain the supplementary crystallographic data for this paper.^23*a*–*g*^ The data supporting this article have been included as part of the supplementary information (SI). Supplementary information: comprehensive experimental details and characterization including the synthesis and NMR spectra of the tetraimidazolium macrocycle and its Ag(i)/Au(i) coordination cages, supporting HR-MS and UV-vis titration data, full single-crystal X-ray diffraction analyses (CCDC 2468088–2468094), and computational details from DFT calculations with Cartesian coordinates. See DOI: https://doi.org/10.1039/d5ra05896a.
