# Supplementary material for: Tetraimidazolium macrocycle: a versatile building block and precursor for box-type coordination cages
Source: RSC Adv. 2025 Oct 2;15(44):36704–9. doi: 10.1039/d5ra05896a (PMC12495399; doi:10.1039/d5ra05896a)
Supplement: RA-015-D5RA05896A-s001 [file RA-015-D5RA05896A-s001.pdf]

Supplementary Information (SI) for RSC Advances.
This journal is © The Royal Society of Chemistry 2025

Tetraimidazolium Macrocycle: A Versatile Building Block and Precursor for Box-Type Coordination Cages

Fang Wang,*[a] Kai Hua[b]

[a]*School of Chemistry and Chemical Engineering, Yan'an University, Yan'an 716000, P.R. China.*

[b]*Center of Basic Molecular Science (CBMS), Department of Chemistry, Tsinghua University, Beijing 100084, China.*

-Supporting Information-

Material and Methods……………………………………………………………..S1

S1. Synthesis and Characterization………………………………………..………S2

S2. NMR Spectral Analysis…………………………………………………...……S5

S3. Mass Spectrometry…………………………………………………………….S8

S4. X-Ray Diffraction Studies…………………………………………………….S9

S5. UV/Vis Spectroscopic Analysis………………………………………………S19

S6 Computational Details………………………………………………………..S22

S7. References……………………………………………………………………..S32

Material and Methods

All starting materials were used as received from commercial sources, while solvents were freshly distilled by standard procedures prior to use. The experiments were carried out under the nitrogen atmosphere with standard Schlenk techniques. 1-(1H-imidazol-1-ylmethyl)-1H-imidazole[1] was synthesized according to reported procedures. All other chemicals were purchased from commercial suppliers were used without further purification. The ^{1}H and ^{13}C{^{1}H} spectra were recorded on Bruker AVANCE III 400 or JEOL JNM-ECZ600R spectrometers. Chemical shifts (δ) are expressed in ppm downfield from tetramethylsilane (TMS) using the residual protonated solvent as an internal standard. All coupling constants are expressed in Hertz. Mass spectra were obtained with a Bruker microTOF-Q II mass spectrometer (Bruker Daltonics Corp., USA) in the electrospray ionization (ESI) mode. UV-Vis spectra were measured using an Agilent Cary-100 spectrophotometer.

S1. Synthesis and Characterization

1.1 Synthesis of H_4-1$(PF_6)_4$

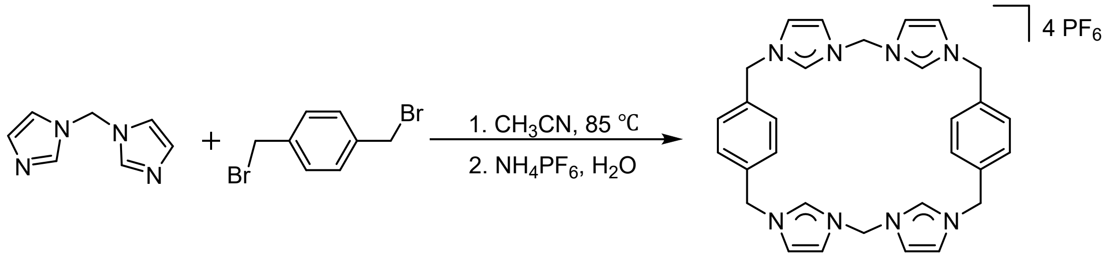

H_4-**1**$(PF_6)_4$

In a typical procedure, a 250 mL round bottom flask was charged with a solution of 1-(1H-imidazol-1-ylmethyl)-1H-imidazole (250.0 mg, 1.69 mmol) in acetonitrile (60 mL). Next, a solution of the appropriate 1, 4-bis (bromomethyl)benzene (446.2 mg, 1.69 mmol) in acetonitrile (50 mL) was added dropwise to the reaction mixture over a period of 4 h. The mixture was heated under reflux for 48 h. Then, the reaction mixture was cooled to room temperature and the solvent was subsequently removed via rotary evaporation. The residue was then dissolved in water (100 mL) and NH_4PF_6 (1.67 g, 10.14 mmol) was added to the solution. This gave rise to a light precipitate, which was filtered off and washed with 100 mL water. The crude product was recrystallized from acetonitrile and water to give out the pure product. Yield: 1.39 g (1.28 mmol, 76%). ^{1}H NMR (400 MHz, DMSO-d_6): δ = 9.49 (d, J = 10.7 Hz, 4H), 7.99 (d, J = 10.7 Hz, 4H), 7.83 (s, 4H,), 7.51 (s, 4H, benzene ring -C-**CH**=**CH**-C-), 7.45, (s, 4H, benzene ring -C-**CH**=**CH**-C-), 6.63 (s, 4H, -N-**CH$_2$**-N-), 6.60, (s, 4H, -N-**CH$_2$**-N-), 5.48 (s, 8H, -N-**CH$_2$**-C-). ^{13}C{^{1}H} NMR (100 MHz, DMSO-d_6): δ = 137.9, 137.6, 134.9, 134.8, 129.3, 129.1, 123.2, 123.1, 122.7, 58.5, 58.2, 51.9. HRMS (ESI, positive ions): m/z = 216.4185 (calcd for $[H_4\text{-}\mathbf{1}(PF_6)]^{3+}$ 216.4125), 397.1032 (calcd for $[H_4\text{-}\mathbf{1}(PF_6)_2]^{2+}$ 397.1011).

Large scale: In a typical procedure, a 1000 mL round bottom flask was charged with a solution of 1-(1H-imidazol-1-ylmethyl)-1H-imidazole (1.0 g, 6.75 mmol) in acetonitrile (300 mL). Next, a solution of the appropriate 1, 4-bis (bromomethyl)benzene (1.78 g, 6.75 mmol) in acetonitrile (200 mL) was added dropwise to the reaction mixture over a period of 8 h. The mixture was heated under

reflux for 48 h. Then, the reaction mixture was cooled to room temperature and the solvent was subsequently removed via rotary evaporation. The residue was then dissolved in water (100 mL) and NH_4PF_6 (6.68 g, 40.56 mmol) was added to the solution. This gave rise to a light precipitate, which was filtered off and washed with 100 mL water. The crude product was recrystallized from acetonitrile and water to give out the pure product. Yield: 5.1 g (4.73 mmol, 70%).

1.2 Synthesis of $[Ag_4(1)_2](PF_6)_4$

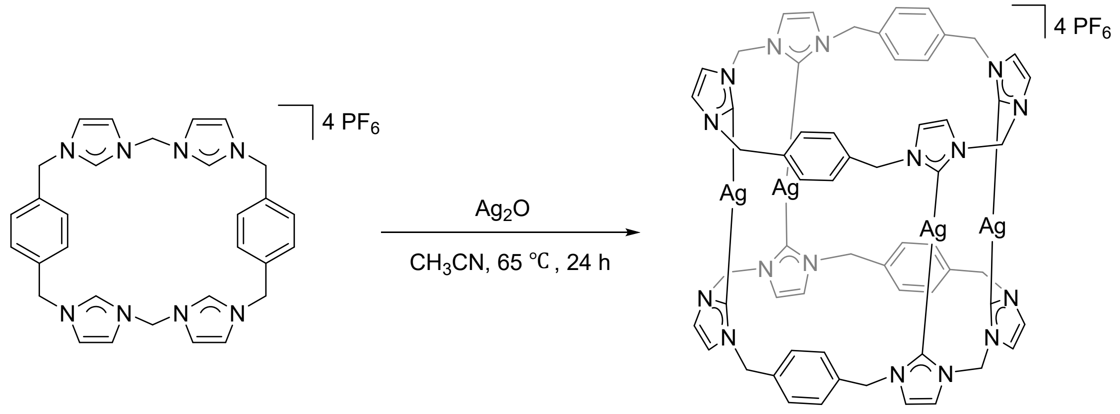

H_4-**1**$(PF_6)_4$ $[Ag_4(\mathbf{1})_2](PF_6)_4$

A sample of H_4-**1**$(PF_6)_4$ (50 mg, 0.046 mmol) was dissolved in 15 mL of CH_3CN and to this solution was added Ag_2O (53 mg, 0.023 mmol). The resulting suspension was heated to 65 °C for 24 h under exclusion of light. After cooling to ambient temperature, the obtained suspension was filtered slowly through Celite to obtain a clear solution. The filtrate was concentrated to 2 mL, and diethyl ether (20 mL) was added. This led to the precipitation of a light brown solid. The solid was collected by filtration, washed with diethyl ether, and dried *in vacuo*. Yield: 40 mg (0.020 mmol, 86%). ^1H NMR (400 MHz, DMSO-d_6): δ = 8.05 (s, 4H, imidazolium ring -N-**CH=CH**-N-), 7.44 (s, 4H, imidazolium ring -N-**CH=CH**-N-), 7.33 (d, J = 13.4 Hz, 2H, imidazolium ring -N-**CH**-N-), 6.89 (s, 4H, benzene ring -C-**CH=CH**-C-), 6.77 (s, 4H, benzene ring -C-**CH=CH**-C-), 6.39 (d, J = 13.4 Hz, 2H, imidazolium ring -N-**CH**-N-), 5.20 (s, 8H, -N-**CH$_2$**-C-). $^{13}C\{^1H\}$ NMR (125 MHz, DMSO-d_6): δ = 181.70, 135.7, 126.4, 123.7, 122.5, 63.3, 53.7. HRMS (ESI, positive ions): m/z = 525.6949 (calcd for $[Ag_4(\mathbf{1})_2(PF_6)]^{3+}$ 525.6902), 861.0232 (calcd for $[Ag_4(\mathbf{1})_2(PF_6)_2]^{2+}$

861.0176), 1866.9933 (calcd for $[Ag_4(\mathbf{1})_2(PF_6)_3]^+$ 1867.0000).

1.3 Synthesis of $[Au_4(1)_2](PF_6)_4$

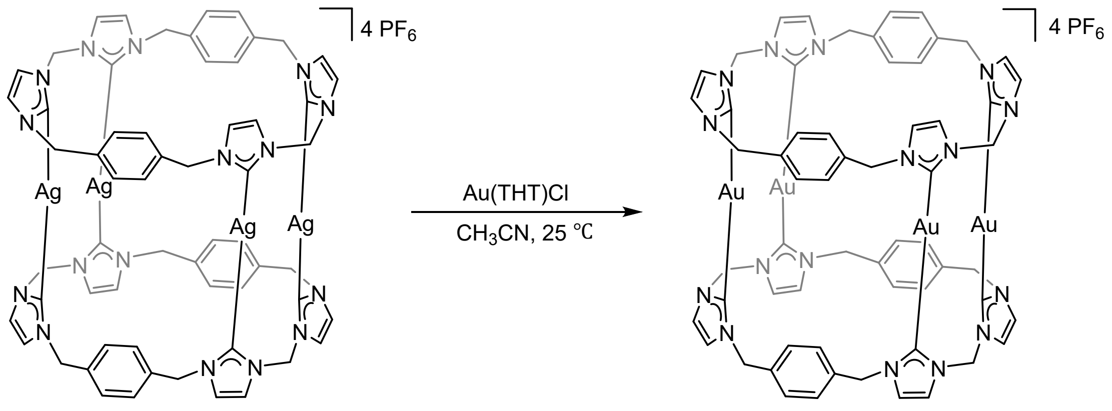

$[Ag_4(\mathbf{1})_2](PF_6)_4$ $[Au_4(\mathbf{1})_2](PF_6)_4$

A solution of $[Ag_4(\mathbf{1})_2](PF_6)_4$ (80 mg, 0.04 mmol) in acetonitrile (15 mL) was treated with solid [AuCl(THT)] (64 mg, 0.20 mmol). During the addition some white and purple solid precipitated. The reaction mixture was stirred at ambient temperature for 24 h and then slowly filtered to get a clear filtrate. The filtrate was added slowly to diethyl ether (25 mL). Upon this addition a white solid precipitated which was collected by filtration, washed with diethyl ether and dried in vacuo without heating to give $[Au_4(\mathbf{1})_2](PF_6)_4$ as a colorless solid. Yield: 60 mg (0.025 mmol, 63%). ^{1}H NMR (400 MHz, CD_3CN): δ = 7.63 (s, 4H, imidazolium ring **-**N**-CH=CH-**N**-**), 7.14 (s, 4H, imidazolium ring **-**N**-CH=CH-**N**-**), 7.11 (d, J = 15.2 Hz, 2H, imidazolium ring **-**N**-CH-**N**-**), 6.88 (s, 8H, benzene ring **-**C**-CH=CH-**C**-**) ,6.18 (d, J = 15.2 Hz, 2H, imidazolium ring **-**N**-CH-**N**-**), 5.13 (dd, $J_{H,H}$ = 16.2 Hz, $J_{H,H}$ = 16.5 Hz, 8H, **-**N**-CH$_2$-**C**-**). HRMS (ESI, positive ions): m/z = 447.0971 (calcd for $[Au_4(\mathbf{1})_2]^{4+}$ 447.0878), 644.4461 (calcd for $[Au_4(\mathbf{1})_2(PF_6)]^{3+}$ 644.4387).

S2. NMR spectral analysis

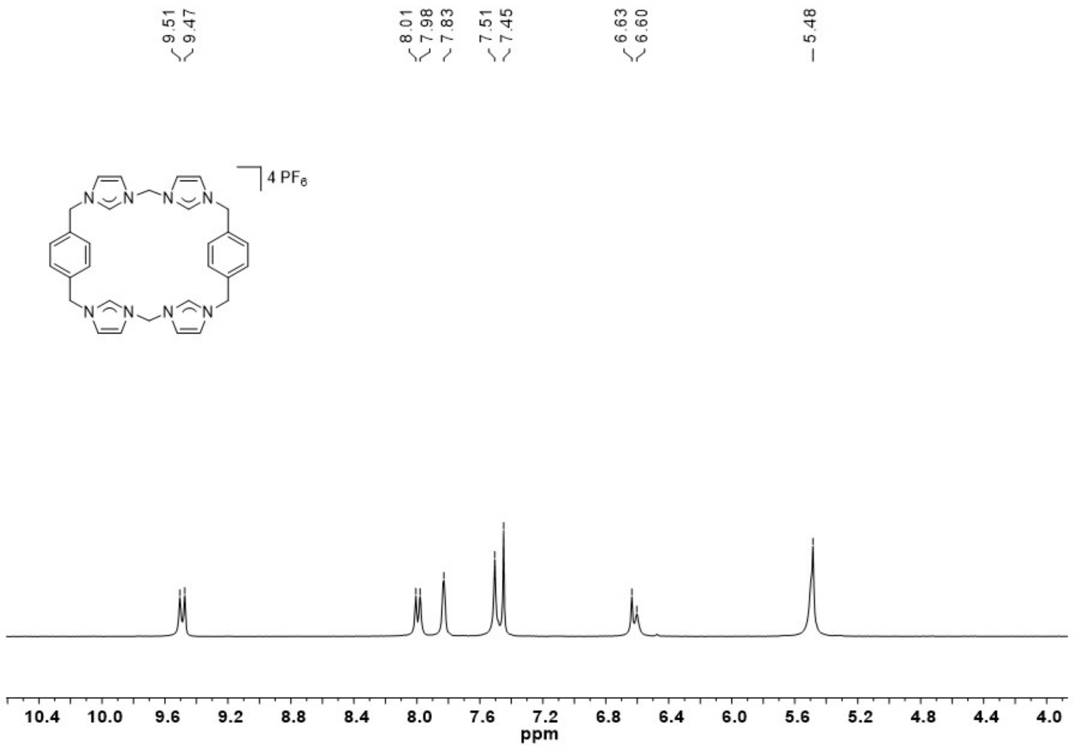

Figure S1. ^{1}H NMR (400 MHz, DMSO-d_6) of H_4-**1**$(PF_6)_4$

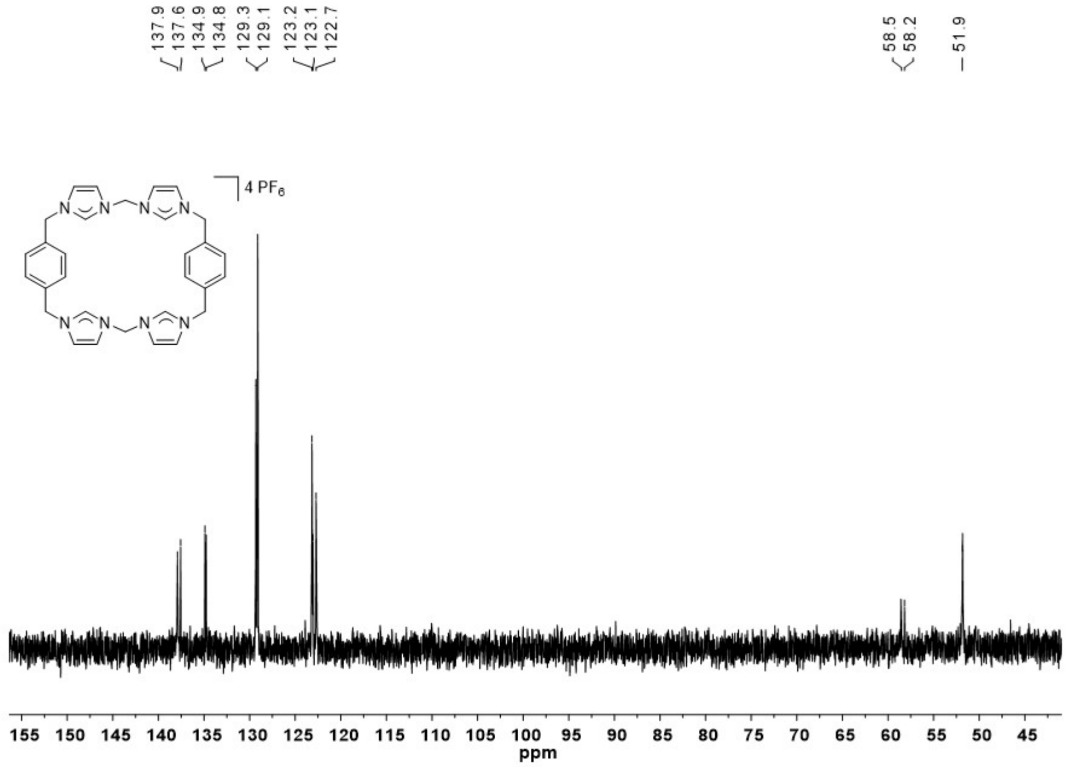

Figure S2. ^{13}C NMR (100 MHz, DMSO-d_6) of H_4-**1**$(PF_6)_4$

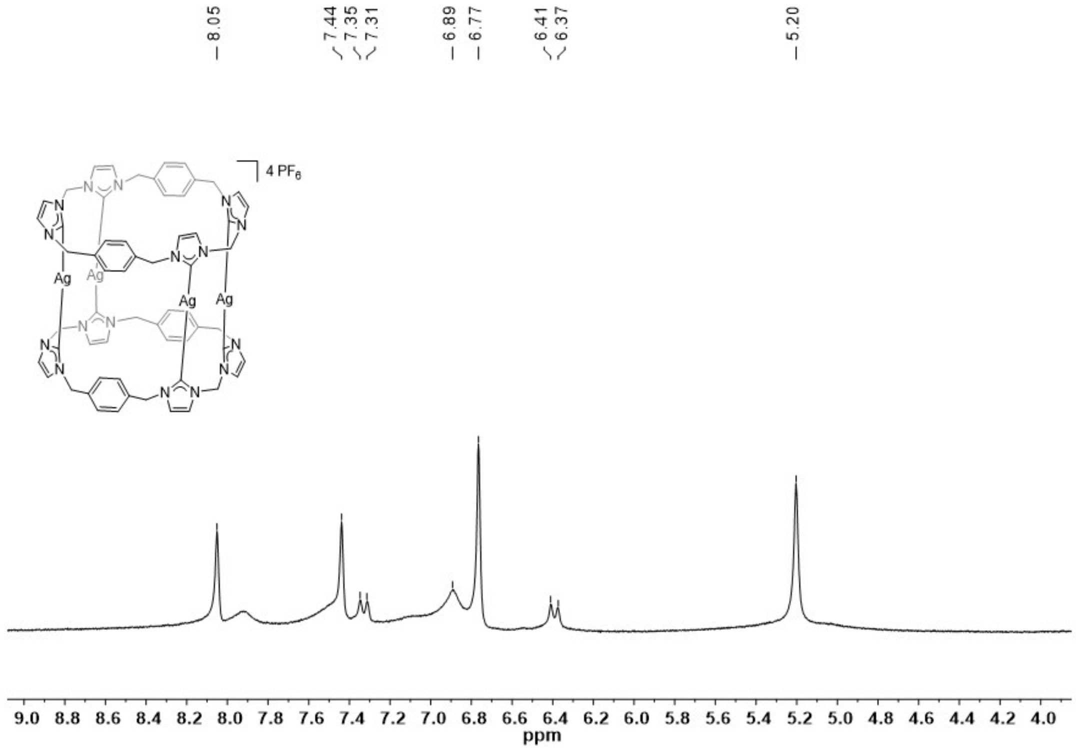

Figure S3. ^{1}H NMR (400 MHz, DMSO-d_6) of $[Ag_4(\mathbf{1})_2](PF_6)_4$

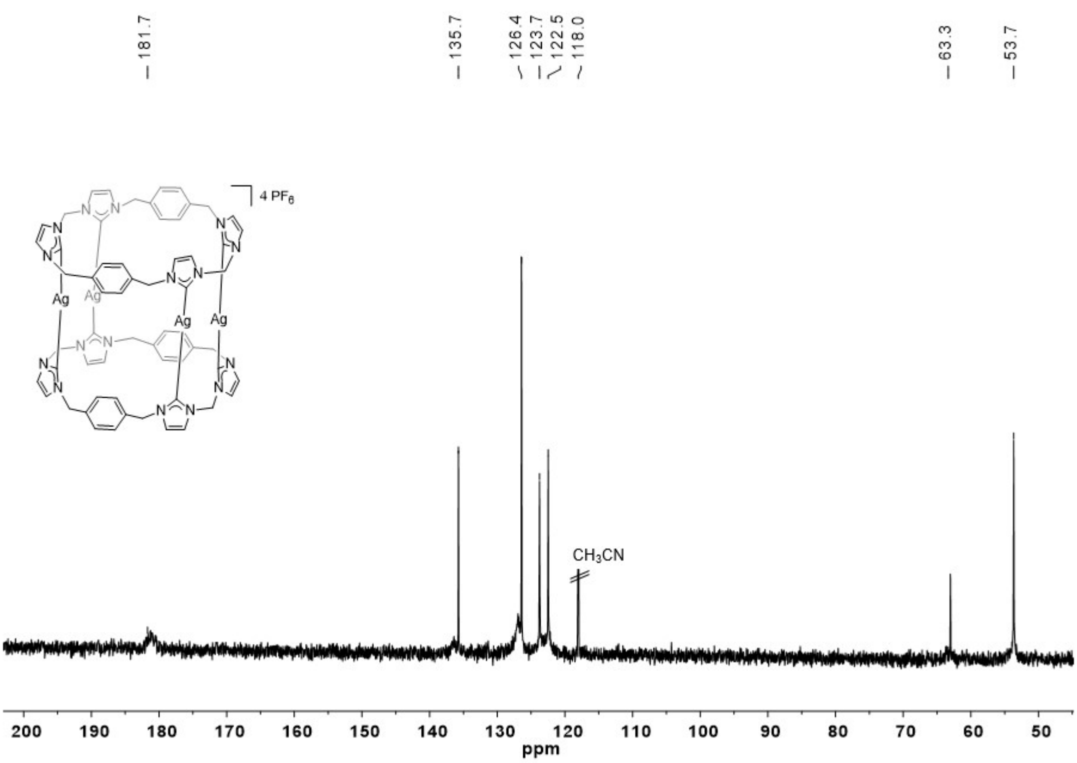

Figure S4. ^{13}C NMR (125 MHz, DMSO-d_6) of $[Ag_4(\mathbf{1})_2](PF_6)_4$

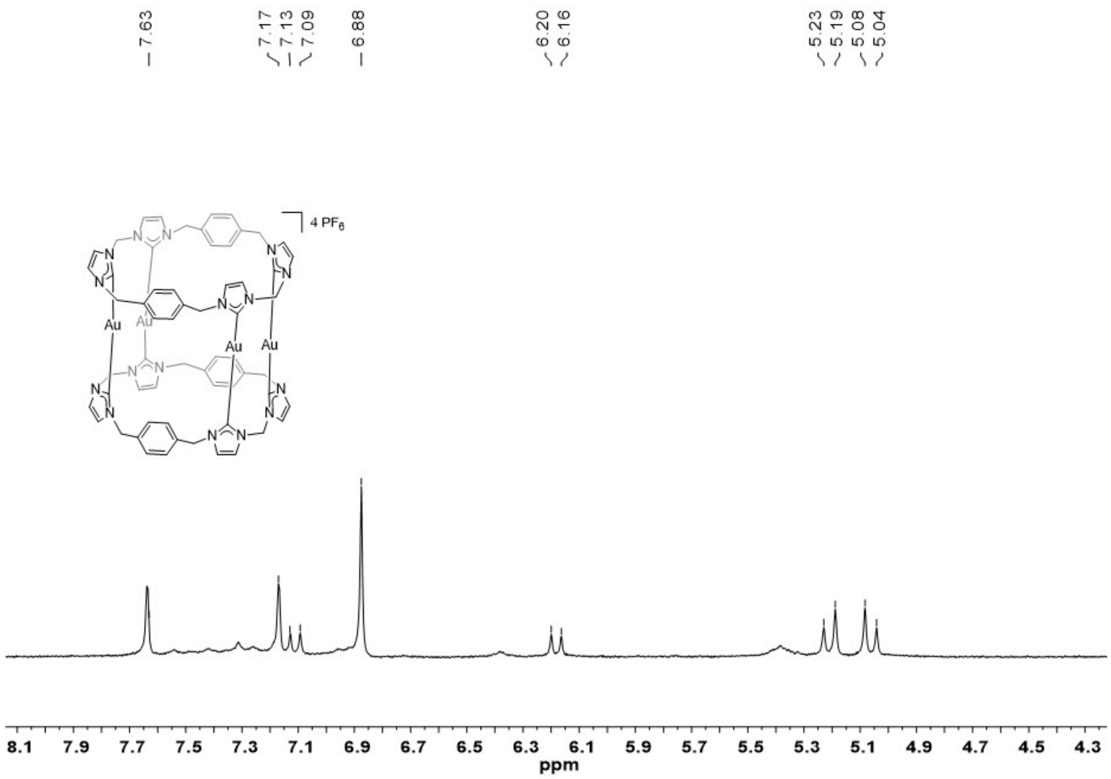

Figure S5. ^{1}H NMR (400 MHz, CD_3CN) of $[Au_4(\mathbf{1})_2](PF_6)_4$

S3. Mass Spectrometry

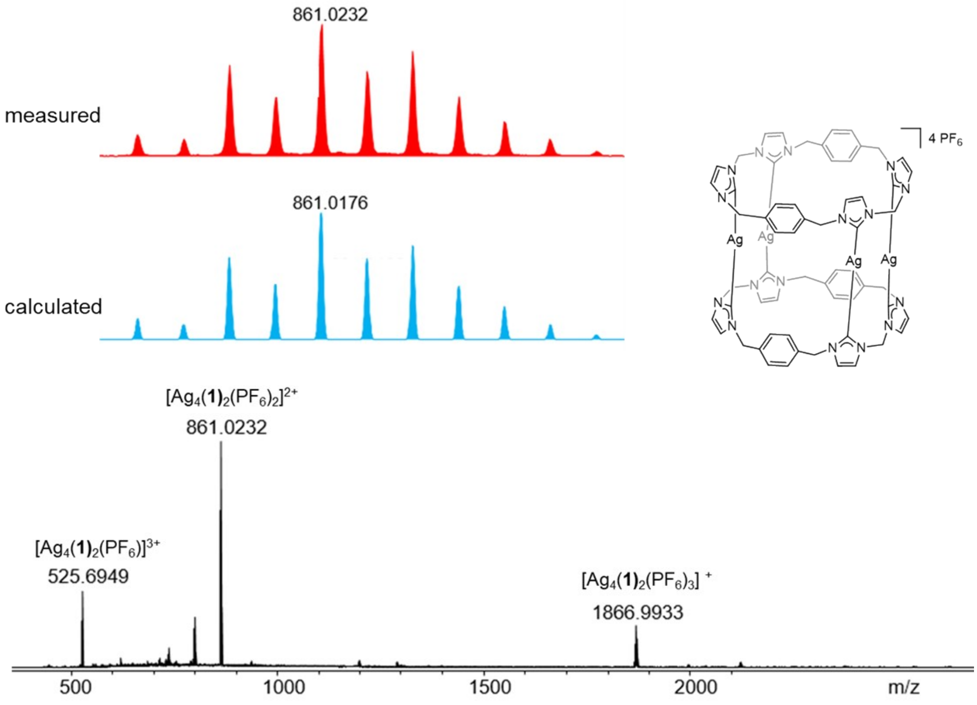

Figure S6. HR-ESI mass spectrum (positive ions) of compound $[Ag_4(\mathbf{1})_2](PF_6)_4$. (experimentally observed distribution on top and simulated distribution at the bottom).

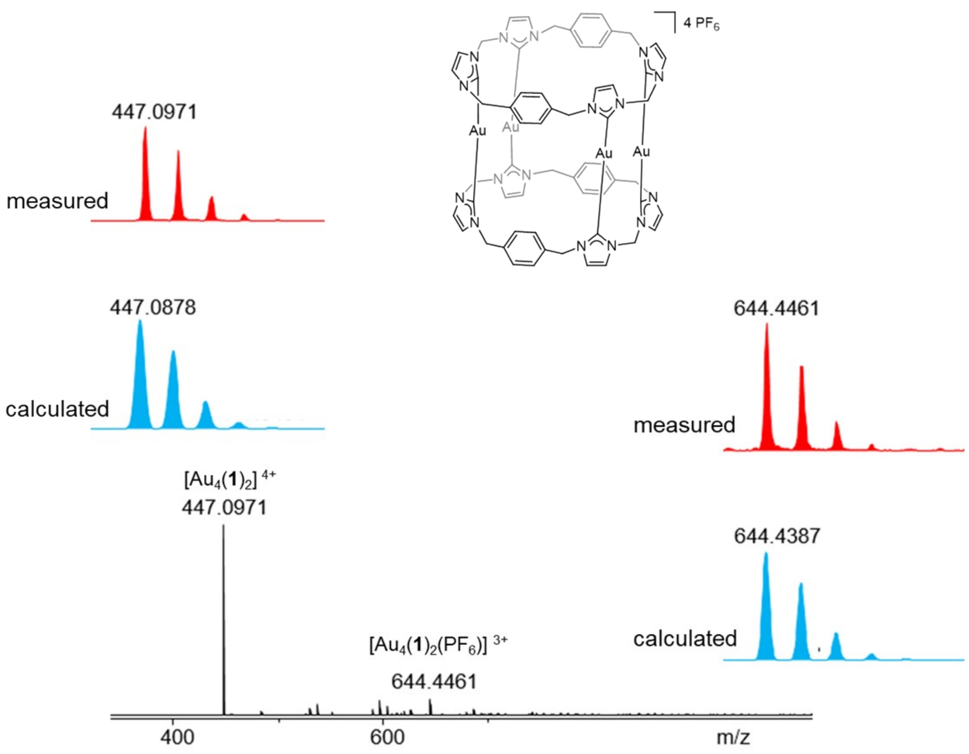

Figure S7. HR-ESI mass spectrum (positive ions) of compound $[Au_4(\mathbf{1})_2](PF_6)_4$. (experimentally observed distribution on top and simulated distribution at the bottom).

S4. X-Ray Diffraction studies

A suitable crystal was selected and performed on a Bruker APEX-II CCD diffractometer equipped with a rotation anode using graphite-monochromated Mo K_α radiation (λ = 0.71073 Å). Raw data collection and processing were performed with APEX II software package. The data were corrected for absorption using the SADABS program.[2] Using Olex2,[3] the structure was solved with the olex2.solve[4] structure solution program using Charge Flipping and refined with the ShelXL[5] refinement package using Least Squares minimisation. The disordered solvent molecules which could not be restrained properly were removed using the SQUEEZE route.[6]

Table S1. Data Collection and Refinement for Crystals Incorporating [(H_4-**1**)(PF_6)$_4$·2CH_3CN·2H_2O], [(H_4-**1**)(1,5-nds)$_2$·2CH_3CN·9H_2O], [(H_4-**1**)(2,6-nds)$_{1.5}$$PF_6$·3$H_2O$] or [($H_4$-**1**)(2,7-nds)$_2$·2$CH_3CN$·5$H_2O$]

identification code	[(H_4-**1**)(PF_6)$_4$·2CH_3CN·2H_2O]	[(H_4-**1**)(1,5-nds)$_2$·2CH_3CN·9H_2O]	[(H_4-**1**)(1,5-nds)$_2$·2CH_3CN·9H_2O]	[(H_4-**1**)(2,7-nds)$_2$·2CH_3CN·5H_2O]
CCDC Number	2468088	2468089	2468090	2468091
empirical formula	$C_{34}H_{42}F_{24}N_{10}O_2P_4$	$C_{54}H_{70}N_{10}O_{21}S_4$	$C_{45}H_{47}F_6N_8O_{12}PS_3$	$C_{54}H_{60}N_{10}O_{17}S_4$
formula weight	1202.66	1323.4	1133.06	1249.36
temperature/K	293(2)	153.04	150.01	166.0
crystal system	monoclinic	triclinic	triclinic	triclinic
space group	*P*2$_1$/c	*P*-1	*P*-1	*P*-1

a/Å	9.7103(14)	9.9900(12)	13.5033(6)	13.1874(10)
b/Å	15.367(2)	11.5725(14)	13.5926(6)	13.5426(9)
c/Å	16.634(2)	13.5773(17)	16.9599(7)	16.6450(11)
α/°	90	106.084(4)	77.3840(10)	103.076(2)
β/°	106.660(2)	93.193(4)	86.6340(10)	93.461(2)
γ/°	90	91.577(4)	61.5670(10)	91.759(2)
volume/Å^3	2377.9(6)	1504.4(3)	2667.6(2)	2887.3(3)
Z	2	1	2	2
ρ_{calc}g/cm^3	1.680	1.461	1.411	1.437
μ/mm^{-1}	0.299	0.244	0.256	0.245
F(000)	1216.0	704.0	1378.0	1308.0
crystal size/mm^3	0.120 × 0.110 × 0.100	0.21 × 0.20 × 0.15	0.25 × 0.20 × 0.15	0.30 × 0.26 × 0.18

radiation	MoKα (λ = 0.71073)	MoKα (λ = 0.71073)	MoKα (λ = 0.71073)	MoKα (λ = 0.71073)
2Θ range for data collection/deg	3.682 to 50.016	4.982 to 50.802	4.636 to 50.77	4.41 to 50.774
index ranges	-11 ≤ h ≤ 11, -18 ≤ k ≤ 12, -18 ≤ l ≤ 19	-12 ≤ h ≤ 11, -13 ≤ k ≤ 12, -16≤ l ≤ 6	-16 ≤ h ≤ 15, -16 ≤ k ≤ 16, -20≤ l ≤ 20	-15 ≤ h ≤ 15, -16 ≤ k ≤ 16, -20 ≤ l ≤ 19
reflections collected	11554	16014	39097	49194
independent reflections	4167 [R_{int} = 0.0246, R_{sigma} = 0.0302]	5515 [R_{int} = 0.0660, R_{sigma} = 0.0737]	9768 [R_{int} = 0.0453, R_{sigma} = 0.0433]	10444 [Rint = 0.0548, Rsigma = 0.0490]
data/restraints / parameters	4167/0/335	5515/108/451	9768/47/713	10444/0/788
goodness-of-fit on F^2	1.084	1.032	1.026	1.052
final R indexes [I ≥ 2σ(I)]	R_1 = 0.0423, wR_2 = 0.1354	R_1 = 0.0685, wR_2 = 0.1772	R_1 = 0.0706, wR_2 = 0.1780	R_1 = 0.0821, wR_2 = 0.2112
final R indexes [all data]	R_1 = 0.0500, wR_2 = 0.1534	R_1 = 0.0868, wR_2 = 0.1944	R_1 = 0.0952, wR_2 = 0.1964	R_1 = 0.0984, wR_2 = 0.2204
largest diff. peak/hole/e $Å^{-3}$	0.41/-0.84	0.64/-1.04	1.44/-0.89	0.97/-0.54

Table S2. Data Collection and Refinement for Crystals Incorporating $[Ag_4(\mathbf{1})_2](BPh_4)_4{\cdot}10CH_3CN$, $[Ag_4(\mathbf{1})_2](BPh_4)_4{\cdot}CH_3CN{\cdot}6DMSO{\cdot}Et_2O$ and $[Au_4(\mathbf{1})_2](BPh_4)_4{\cdot}2CH_3CN{\cdot}6DMSO{\cdot}Et_2O$

identification code	$[Ag_4(\mathbf{1})_2](BPh_4)_4{\cdot}10CH_3CN$	$[Ag_4(\mathbf{1})_2](BPh_4)_4{\cdot}CH_3CN{\cdot}6DMSO{\cdot}Et_2O$	$[Au_4(\mathbf{1})_2](BPh_4)_4{\cdot}2CH_3CN{\cdot}6DMSO{\cdot}Et_2O$
CCDC Number	2468092	2468093	2468094
empirical formula	$C_{176}H_{166}Ag_4B_4N_{26}$	$C_{174}H_{185}Ag_4B_4N_{17}O_7S_6$	$C_{176}H_{188}Au_4B_4N_{18}O_7S_6$
formula weight	3120.06	3293.47	3690.92
temperature/K	153.0	116.29	114.28
crystal system	triclinic	triclinic	triclinic
space group	P-1	P-1	P-1
a/Å	14.7897(5)	14.6113(6)	14.6171(10)
b/Å	16.4560(5)	16.2446(7)	16.3251(11)
c/Å	17.2188(6)	18.3871(8)	18.3596(12)
α/°	83.5650(10)	83.3440(10)	83.429(2)
β/°	75.8980(10)	76.497(2)	76.404(2)
γ/°	69.6420(10)	69.0310(10)	69.016(2)
volume/$Å^3$	3808.8(2)	3960.1(3)	3973.7(5)
Z	1	1	1
$\rho_{calc}g/cm^3$	1.360	1.381	1.542
μ/mm^{-1}	0.567	0.630	3.824
F(000)	1612.0	1708.0	4044.0
crystal size/mm^3	0.20 × 0.18 × 0.15	0.25 × 0.20 × 0.15	0.25 × 0.2 × 0.18
radiation	MoKα (λ = 0.71073)	MoKα (λ = 0.71073)	MoKα (λ = 0.71073)

2Θ range for data collection/deg	4.62 to 50.71	4.56 to 50.806	4.176 to 50.794
index ranges	-16 ≤ h ≤ 17, -19 ≤ k ≤ 19, -20 ≤ l ≤ 20	-17 ≤ h ≤ 17, -19 ≤ k ≤ 19, -22 ≤ l ≤ 22	-17 ≤ h ≤ 17, -19 ≤ k ≤ 19, -22 ≤ l ≤ 22
reflections collected	47222	65078	97650
independent reflections	13902 [R_{int} = 0.0486, R_{sigma} = 0.0491]	14546 [R_{int} = 0.0539, R_{sigma} = 0.0440]	14569 [R_{int} = 0.0503, R_{sigma} = 0.0273]
data/restraints/ parameters	13902/57/986	14546/56/1020	14569/246/1089
goodness-of-fit on F^2	1.036	1.025	1.093
final R indexes [I ≥ 2σ(I)]	R_1 = 0.0377, wR_2 = 0.0836	R_1 = 0.0670, wR_2 = 0.1754	R_1 = 0.0308, wR_2 = 0.0892
final R indexes [all data]	R_1 = 0.0539, wR_2 = 0.0927	R_1 = 0.0846, wR_2 = 0.1880	R_1 = 0.0379, wR_2 = 0.0948
largest diff. peak/hole/e $Å^{-3}$	1.07/-0.47	4.65/-3.39	1.90/-1.61

Single-crystal analysis for structure [(H_4-**1**)(1,5-nds)$_2$·2CH_3CN·9H_2O]**:**

To a solution of 3.4 mg (H_4-**1**)(PF_6)$_4$ (2.8 mM in acetonitrile) in 1.5 mL acetonitrile was added 5.5 molar equivalents of disodium 1,5-naphthalenedisulfonate (1,5-nds) dissolved in water. A white solid precipitated from the initially clear solution. Additional acetonitrile was added to redissolve the precipitate and restore clarity. The clear solution was filtered and then allowed to evaporate slowly at room temperature. After approximately one week, colorless prismatic crystals of [(H_4-**1**)(1,5-nds)$_2$] formed. These crystals were suitable for single-crystal X-ray diffraction analysis. The complex crystallizes in the triclinic space group *P* 1. The asymmetric unit consists of one (H_4-**1**)$^{4+}$ cation, two 1,5-nds anions, two acetonitrile molecules, and nine water molecules. The single-crystal structure revealed that the complexation between the (H_4-1)$^{4+}$ macrocycle and the 1,5-nds anions adopts an 'outside' binding mode (Figure S8).

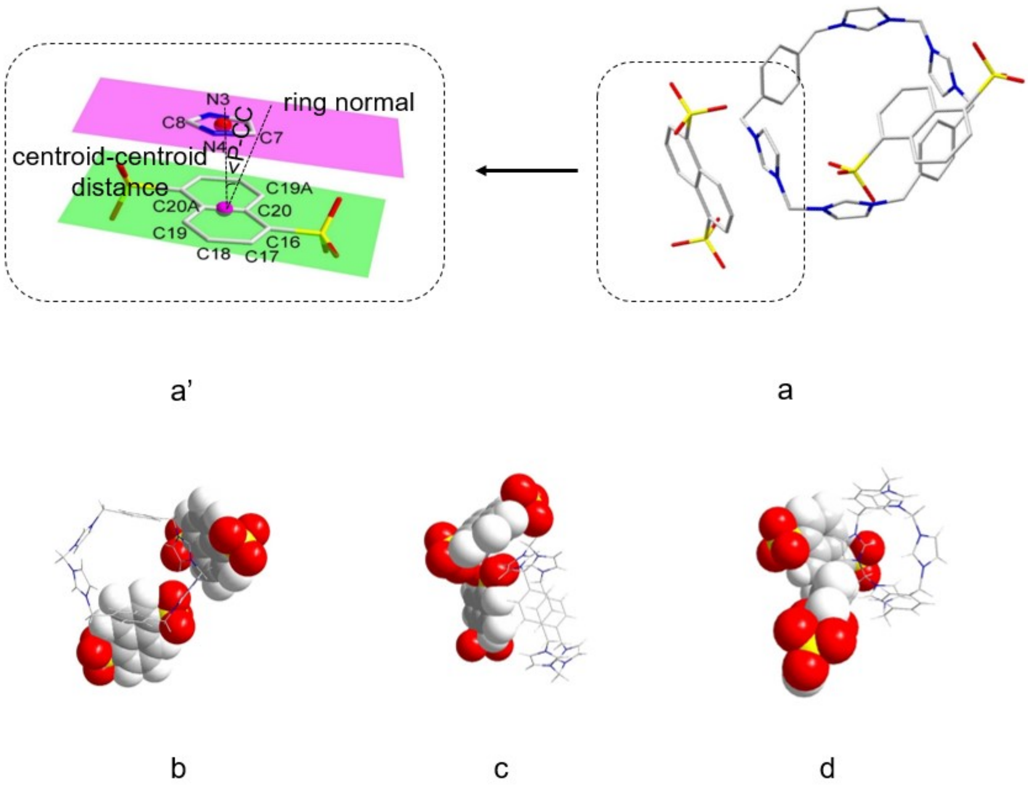

Figure S8. Top view in sticks form (a) showing the binding mode between (H_4-**1**)$^{4+}$ and 1,5-nds in the single-crystal structure of (H_4-**1**)$^{4+}$ and expanded π-π donor-acceptor interaction stacking part (a’) in stick form. Also shown as a top view (b),

side view (c) and front view (d) of the structure [(H_4-**1**)(1,5-nds)$_2$] in stick-spacefill form. All the other molecules and atoms have been omitted for clarity. The possible π-π interactions were inferred from the following selected interatomic distances [Å]: C(8)···C(20A) 3.727(4), N(4)···C(19) 3.744(0), C(7)···C(20) 3.728(4), C(7)···C(16) 3.854(4)

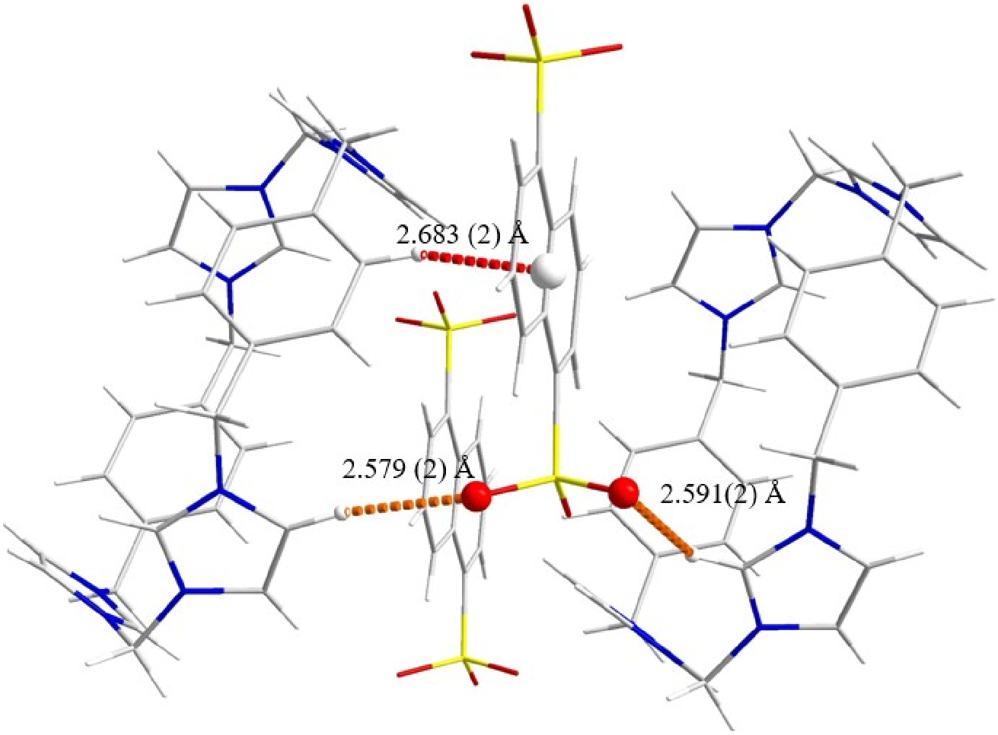

Figure S9. Top view in sticks form of (H_4-**1**)$^{4+}$ and 1,5-nds.

Single-crystal analysis for structure [(H_4-**1**)(2,6-nds)$_{1.5}$$PF_6$·3$H_2O$]**:**

solution of 3.4 mg (2.8 mM) of (H_4-**1**)(PF_6)$_4$ in 1.5 mL acetonitrile was treated with 5.5 molar equivalents of disodium 2,6-naphthalenedisulfonate (2,6-nds) in water, resulting in the precipitation of a white solid from the initially clear solution. Additional acetonitrile was added to redissolve the precipitate and restore clarity. Following filtration, slow evaporation of the clear solution yielded colorless prismatic crystals of [(H_4-**1**)(2,6-nds)$_2$] over approximately one week; these crystals were used for X-ray diffraction analysis.

The complex [(H_4-**1**)(2,6-nds)$_2$] crystallizes in the triclinic space group *P*-1. The asymmetric unit contains two (H_4-**1**)$^{4+}$ cations, 1.5 2,6-nds anions, one PF_6^- anion, and three water molecules. External binding modes were observed between (H_4-**1**)$^{4+}$ and

neighboring complexes within the crystal lattice (Figure S9). Additionally, a 2,6-nds anion resides outside the cavity of a neighboring (H_4-**1**)$^{4+}$ cation, stabilized by π-π donor-acceptor interactions involving the benzene ring (specifically atom C30) of (H_4-**1**)$^{4+}$ and the naphthalene plane of 2,6-nds. Key geometric parameters for this interaction are the centroid-centroid distance (C···C) = 3.694 Å and the P-CC displacement angle (θ, defined as the angle between the centroid vector and the normal to the 2,6-naphthalenedisulfonate ring plane) = 27.670°.

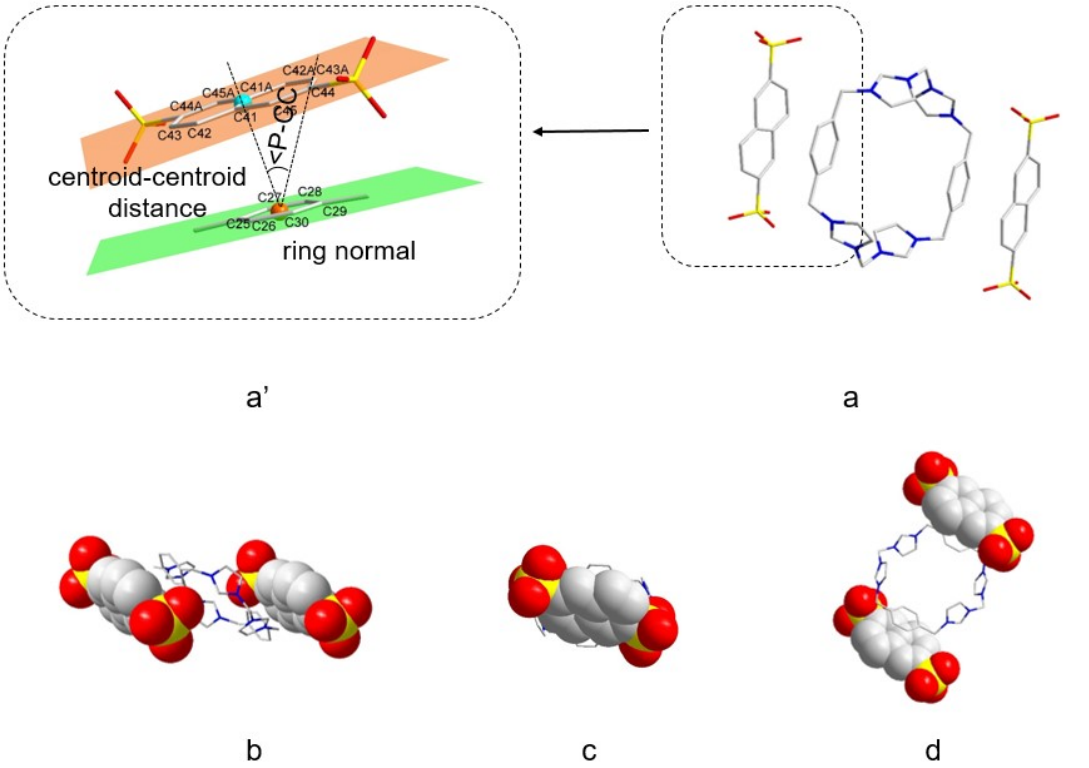

Figure S10. Top view in sticks form (a) showing the binding mode between (H_4-**1**)$^{4+}$ and 2,6-naphthalene disulfonate dianion (2,6-nds) in the single-crystal structure of (H_4-**1**)$^{4+}$ and expanded π-π donor-acceptor interaction stacking part (a') in stick form. Also shown as a top view (b), side view (c) and front view (d) of the structure [(H_4-**1**)(2,6-nds)$_{1.5}$$PF_6$] in stick-spacefill form. All the other molecules and atoms have been omitted for clarity. The possible π-π interactions were inferred from the following selected interatomic distances [Å]: C(25)···C(42) 3.753(1), C(26)···C(41) 3.537(1), C(27)···C(45A) 3.900(1), C(28)···C(42A) 3.938(1), C(29)···C(44) 3.891(1), C(30)···C(45) 3.515(1).

Single-crystal analysis for structure [(H_4-**1**)(2,7-nds)$_2$·2CH_3CN·5H_2O]**:**

Addition of 5.5 molar equiv of disodium 2,7-naphthalenedisulfonate (2,7-nds) in water to a solution of 3.4 mg (H_4-**1**)(PF_6)$_4$·(2.8 mM) in 1.5 ml acetonitrile. White solid precipitated from the clear solution. Some acetonitrile was added to make the solution clear. Evaporating the clear solution slowly after filtering, colorless prisms of [(H_4-**1**)(2,7-nds)$_2$] were obtained from the solution in about one week. These crystals were used to determine the structure of the resulting complexes *via* X-ray diffraction analysis. The complex [(H_4-**1**)(2,7-nds)$_2$] crystallizes in triclinic space group *P*-1. Two molecule of 2,7-nds, six H_2O construct one unit cell. The 2,7-nds outside the (H_4-**1**)$^{4+}$ such that the oxygen atoms is farther than 3.5 Å from its neighboring aromatic systems. Besides, the hydrogen bonds between CH of (H_4-**1**)$^{4+}$ and oxygen atoms of 2,7-nds is shorted 3.0 Å. Such a finding leads us to suggest that the complex is stabilized in part by anion-π interactions and hydrogen bond.

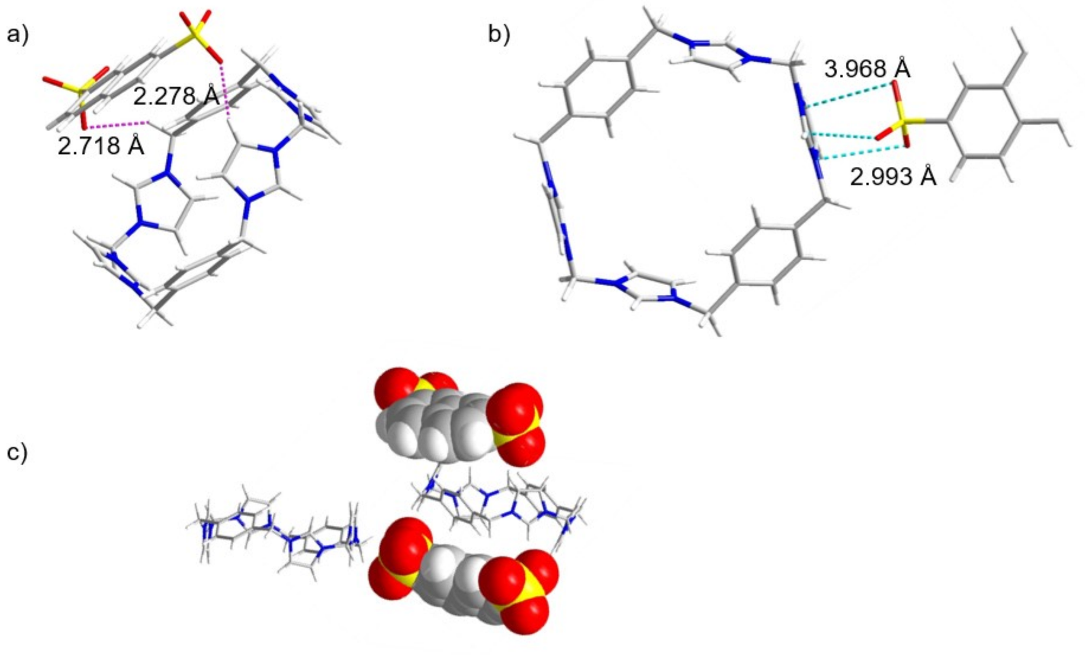

Figure S11. Top view in sticks form (a) showing the hydrogen bonds between (H_4-**1**)$^{4+}$ and oxygen atoms of 2,7-naphthalene disulfonate dianion as pink dashed lines, (b) The anions-π interactions were denoted as turquiose dashed lines, (c) Molecular structure of (H_4-**1**)$^{4+}$ and 2,7-nds. The acetonitrile and H_2O molecules are omitted for clarity.

S5. UV/Vis Spectroscopic Analysis

An additional UV-vis spectroscopic study was carried out. Compared with (H_4-**1**)(PF_6)$_4$, the absorbance of complex (H_4-**1**)(nds)$_2$ is no charge-charge transfer band apparent in the long wavelength spectral region.

(a)

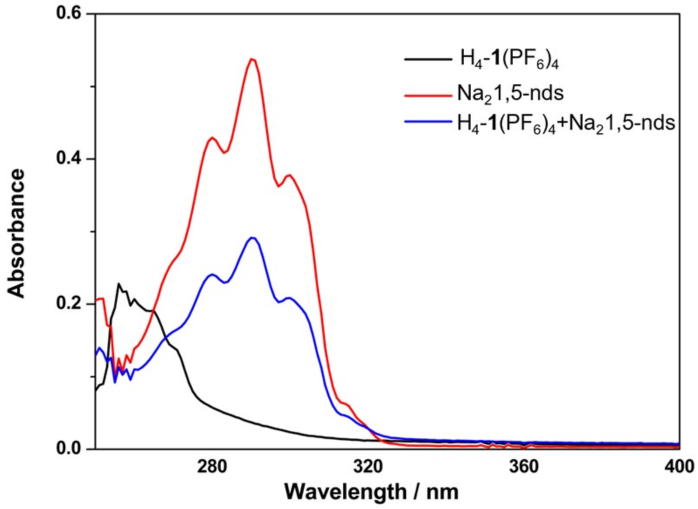

(b)

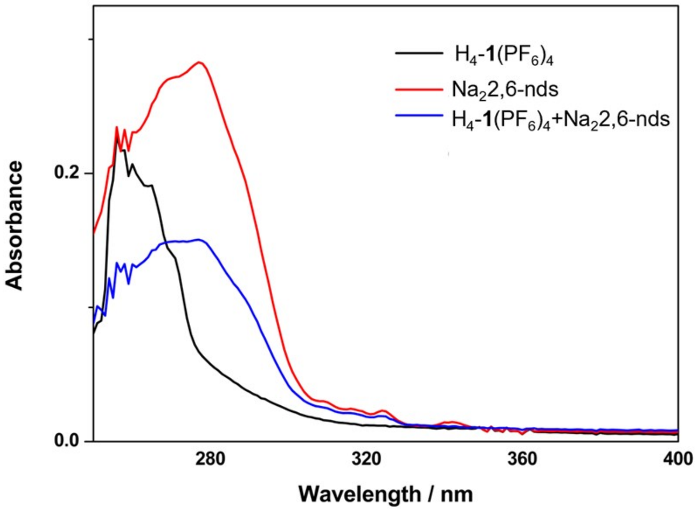

(c)

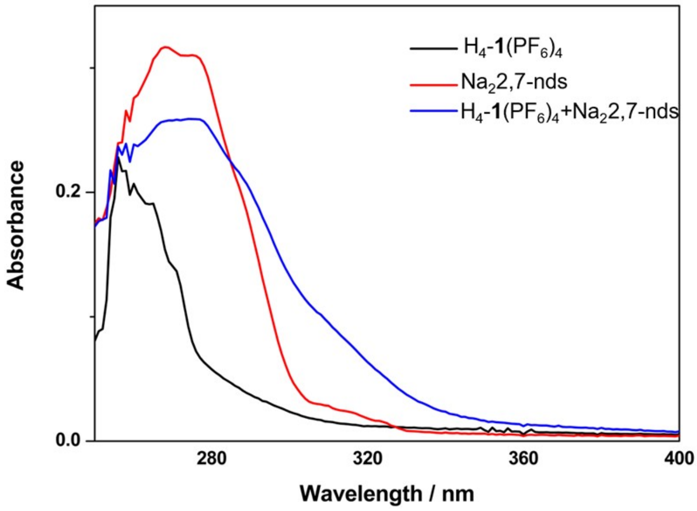

Figure S12. UV-vis spectra of $(H_4$**-1)**$(PF_6)_4$ (6.0×10^{-5} M), Na_2nds (6.0×10^{-5} M), and $(H_4$**-1)**$(PF_6)_4$ (6.0×10^{-5} M) in the presence of 1 molar equiv. of 1,5-nds (a), 2,6-nds (b), or 2,7-nds (c) recorded using a 0.5 cm optical length in DMSO.

In the UV-Vis titration experiments, a solution of acetonitrile in DMSO was gradually added to a DMSO solution of the host assembly (5×10^{-5} M). The resulting absorption changes were monitored and subjected to kinetic analysis, which revealed a highly linear correlation consistent with a 1:1 binding model. From this fit, an association constant of 2.98×10^3 M^{-1} was determined for the binding of acetonitrile.

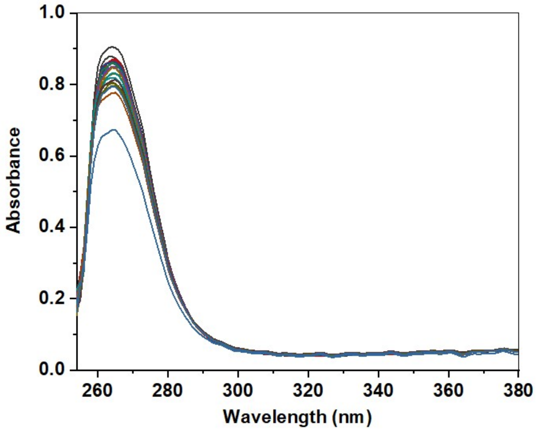

Figure S13. The absorption spectra of Ag_4(**1**)$_2(PF_6)_4$ (5×10^{-5} M) in the absence and

presence of various concentrations of CH_3CN in DMSO at room temperature. The concentrations of CH_3CN ($\times 10^{-5}$ M) were 0, 0.5, 1, 1.5, 2, 2.5, 3, 3.5, 4, 4.5, 5, 6, 7, 8, 9, 10, 15, 20, 25, 50, 100, 150, and 250, respectively.

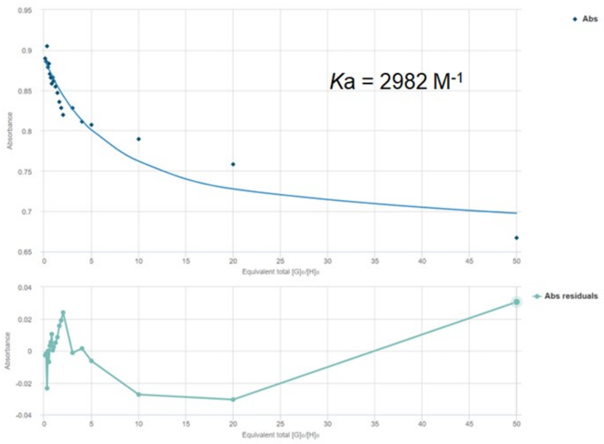

Figure S14. The changes in the UV spectral signal (ΔAbs) of the host upon guest titration were plotted against the [G]/[H] ratio and fitted (lines) using the BindFit program. The association constant is calculated to be Ka = 2982 M^{-1}.

S6. Computational Details

All calculations were performed with the Gaussian(R) 09 program optimizer.[7] The theoretical approach is based on the framework of density functional theory (DFT).[8-9] The geometry optimizations were performed at B3LYP level using LANL2DZ basis set for Ag element and the 6-31G* basis set for all of the other atoms.

Table S3. Cartesians coordinates of calculated $[Ag_4(\mathbf{1})_2]^{4+}$ with CH_3CN at the B3LYP/LANL2DZ (for Ag element) and B3LYP/6-31G* (for other atoms) level of theory.

Atom	X	Y	Z
Ag	4.12849	2.02064	0.15192
Ag	-4.15684	1.87861	-0.11698
N	-4.58602	-1.3066	-3.18754
N	-4.62658	1.13287	-3.19656
N	5.03253	-1.15928	-2.86674
N	4.89906	1.27934	-2.86489
N	3.72916	3.08136	-2.77324
N	3.96067	-3.02151	-2.80966
N	-3.54707	2.98004	-3.00464
N	-3.40217	-3.0894	-2.99924
C	4.24766	2.10159	-1.98945
C	-4.08694	1.97598	-2.26691
C	-1.35174	4.1278	-2.54847
C	-3.94704	-2.08408	-2.26375
C	-1.13353	-4.08906	-2.43383
C	4.35122	-1.98717	-2.01951
C	1.46915	4.16631	-2.47062
C	5.67002	0.0987	-2.48762
H	5.8011	0.10884	-1.40733
H	6.65067	0.14855	-2.96688
C	-5.34728	-0.09641	-2.8874

H	-6.27546	-0.11785	-3.46345
H	-5.58475	-0.09377	-1.82534
C	1.70101	-4.0923	-2.38947
C	-3.71367	-2.95841	-4.34786
H	-3.41456	-3.68606	-5.08742
C	2.97565	4.25187	-2.29286
H	3.3738	5.13143	-2.80649
H	3.23147	4.36362	-1.23422
C	5.08236	-1.68448	-4.15402
H	5.61867	-1.21344	-4.9634
C	-4.46052	-1.83517	-4.46869
H	-4.9353	-1.40133	-5.3352
C	-0.64655	5.30288	-2.83064
H	-1.18232	6.21632	-3.0758
C	4.05034	2.88476	-4.10898
H	3.74604	3.5701	-4.88577
C	3.21737	-4.21493	-2.36477
H	3.56084	-4.45506	-1.3562
H	3.54183	-5.03857	-3.00975
C	0.74939	5.32254	-2.79135
H	1.27281	6.25072	-3.00609
C	-2.8686	4.16595	-2.46104
H	-3.18813	4.24838	-1.41728
H	-3.26179	5.04261	-2.98362
C	-2.64965	-4.23312	-2.45984
H	-2.92924	-5.10407	-3.0634
H	-3.02553	-4.41837	-1.45151
C	4.79535	1.75487	-4.16963
H	5.27602	1.2809	-5.01127

C	-4.43612	1.61872	-4.48814
H	-4.82607	1.12897	-5.36692
C	0.76306	2.98183	-2.22706
H	1.30517	2.06736	-2.00193
C	-3.7482	2.77858	-4.36398
H	-3.41038	3.48084	-5.1111
C	-0.62967	2.96185	-2.26428
H	-1.15804	2.0362	-2.05588
C	4.40443	-2.85676	-4.11546
H	4.23323	-3.59481	-4.88488
C	0.99974	-3.53689	-3.46973
H	1.53692	-3.11565	-4.31529
C	-0.39682	-3.53488	-3.49118
H	-0.90589	-3.11193	-4.35272
C	-0.43168	-4.63403	-1.35279
H	-0.9699	-5.07859	-0.52018
C	0.96407	-4.6372	-1.3319
H	1.475	-5.09024	-0.48657
Ag	-4.02652	-1.94111	-0.11089
Ag	4.17641	-1.86376	0.1252
N	4.58303	1.30937	3.23425
N	4.61848	-1.1288	3.22049
N	-4.95198	1.17891	2.90544
N	-4.9224	-1.26229	2.90293
N	-3.77549	-3.07563	2.81122
N	-3.85662	3.02349	2.79887
N	3.51031	-2.95542	2.99242
N	3.42425	3.10601	3.00877
C	-4.25524	-2.07251	2.03007

C	4.07038	-1.9474	2.27439
C	1.30966	-4.09671	2.53069
C	4.01546	2.1182	2.29105
C	1.19331	4.18215	2.54033
C	-4.33285	2.01695	2.02293
C	-1.51316	-4.14882	2.4825
C	-5.64901	-0.04844	2.54042
H	-5.79944	-0.048	1.46263
H	-6.62158	-0.06372	3.03841
C	5.35236	0.1039	2.94856
H	6.26016	0.11202	3.55656
H	5.6276	0.11396	1.89572
C	-1.62612	4.15532	2.47474
C	3.61894	2.92867	4.37113
H	3.23989	3.62263	5.10614
C	-3.02381	-4.23953	2.32928
H	-3.40768	-5.12065	2.85159
H	-3.2916	-4.36042	1.2743
C	-4.87091	1.66989	4.20674
H	-5.3359	1.18533	5.05106
C	4.3566	1.80169	4.51689
H	4.75858	1.3408	5.40586
C	0.61618	-5.30591	2.66859
H	1.16128	-6.23746	2.79959
C	-4.13654	-2.90393	4.14145
H	-3.86825	-3.61012	4.91267
C	-3.13607	4.20759	2.30876
H	-3.40056	4.30811	1.25117
H	-3.54846	5.08119	2.82144

C	-0.77923	-5.33117	2.64511
H	-1.29352	-6.28234	2.75905
C	2.82592	-4.12602	2.42767
H	3.13724	-4.18083	1.37904
H	3.22682	-5.0158	2.92211
C	2.70676	4.26144	2.44381
H	3.07828	5.15836	2.94742
H	3.02337	4.33139	1.39802
C	-4.86684	-1.76548	4.20043
H	-5.37111	-1.30223	5.03419
C	4.41225	-1.63339	4.50135
H	4.80459	-1.16387	5.3901
C	-0.82105	-2.93587	2.38432
H	-1.36555	-2.00257	2.28074
C	3.70677	-2.78021	4.35506
H	3.35165	-3.48828	5.08863
C	0.57457	-2.91159	2.40824
H	1.08933	-1.95728	2.33464
C	-4.17232	2.82772	4.137
H	-3.8977	3.53221	4.90747
C	-0.89652	2.99071	2.20065
H	-1.40974	2.06862	1.94553
C	0.49635	3.0062	2.23339
H	1.04188	2.09229	2.01146
C	0.46344	5.33444	2.85119
H	0.97913	6.25488	3.11294
C	-0.93279	5.32076	2.81863
H	-1.47714	6.23112	3.05644
C	-0.59683	-0.32155	-0.3112

N	-1.51557	0.07833	0.27561
C	0.54769	-0.84693	-1.04498
H	0.70601	-1.89619	-0.77983
H	1.45053	-0.2777	-0.80233
H	0.36723	-0.7896	-2.12209

Table S4. Cartesians coordinates of calculated $[Ag_4(\mathbf{1})_2]^{4+}$ at the B3LYP/LANL2DZ (for Ag element) and B3LYP/6-31G* (for other atoms) level of theory.

Atom	X	Y	Z
Ag	4.20678	1.89438	7.26E-04
Ag	-4.20597	1.89576	6.22E-04
N	-4.8061	-1.21611	-3.07592
N	-4.80582	1.22008	-3.07518
N	4.80534	-1.21726	-3.07631
N	4.80616	1.21893	-3.07523
N	3.64895	3.01792	-2.87291
N	3.64766	-3.01611	-2.87551
N	-3.64798	3.0187	-2.87323
N	-3.64903	-3.01533	-2.87497
C	4.20482	2.01609	-2.14417
C	-4.20422	2.01724	-2.14429
C	-1.41012	4.10527	-2.45281
C	-4.20499	-2.0141	-2.14546
C	-1.4115	-4.10278	-2.45525
C	4.20413	-2.01525	-2.1459
C	1.4113	4.1049	-2.45258
C	5.55202	0.000465	-2.77977
H	5.80499	-0.000104	-1.72127
H	6.47265	0.000412	-3.368
C	-5.5522	0.002	-2.7795

H	-6.47286	0.00228	-3.36767
H	-5.80511	0.00169	-1.72098
C	1.40995	-4.10308	-2.45556
C	-3.89858	-2.8548	-4.23097
H	-3.55624	-3.56157	-4.97173
C	2.92338	4.17237	-2.32149
H	3.31025	5.07024	-2.81165
H	3.21363	4.23834	-1.26803
C	4.63338	-1.72581	-4.36079
H	5.06872	-1.27192	-5.23765
C	-4.63457	-1.72498	-4.36033
H	-5.06996	-1.27115	-5.2372
C	-0.69733	5.27307	-2.74478
H	-1.22723	6.19556	-2.96785
C	3.89868	2.85854	-4.22901
H	3.5565	3.56598	-4.96922
C	2.92207	-4.17105	-2.32509
H	3.21281	-4.2383	-1.27183
H	3.30851	-5.06844	-2.81646
C	0.69885	5.27288	-2.74467
H	1.22903	6.19524	-2.96765
C	-2.92222	4.17315	-2.32202
H	-3.21268	4.23953	-1.26863
H	-3.30877	5.07096	-2.81254
C	-2.92361	-4.17035	-2.32448
H	-3.31037	-5.06773	-2.81561
H	-3.21417	-4.23734	-1.27116
C	4.63477	1.72888	-4.35922
H	5.07032	1.27584	-5.23643

C	-4.63422	1.7297	-4.35929
H	-5.06991	1.27661	-5.2364
C	0.69729	2.92621	-2.19854
H	1.23039	2.00101	-1.99544
C	-3.89775	2.85913	-4.22931
H	-3.55527	3.56628	-4.96965
C	-0.69647	2.9264	-2.19866
H	-1.22985	2.00134	-1.99562
C	3.897	-2.85538	-4.23153
H	3.55425	-3.56187	-4.97237
C	0.69626	-2.92459	-2.19975
H	1.2296	-1.99978	-1.99547
C	-0.69749	-2.92444	-2.19959
H	-1.23062	-1.99954	-1.99518
C	-0.69903	-5.27035	-2.74892
H	-1.22918	-6.19244	-2.97309
C	0.69716	-5.2705	-2.74907
H	1.22705	-6.1927	-2.97335
Ag	-4.20684	-1.89444	-0.000455
Ag	4.20593	-1.89572	-0.000888
N	4.80632	1.21576	3.07608
N	4.80561	-1.22044	3.07503
N	-4.8051	1.21761	3.07615
N	-4.80635	-1.21857	3.0754
N	-3.64942	-3.01776	2.87333
N	-3.64717	3.01627	2.8751
N	3.64752	-3.01886	2.87281
N	3.64952	3.01518	2.87537
C	-4.20509	-2.01593	2.14445

C	4.20396	-2.01741	2.14402
C	1.40958	-4.10529	2.45252
C	4.20526	2.01392	2.14574
C	1.41207	4.10276	2.45553
C	-4.20385	2.01543	2.14563
C	-1.41184	-4.10488	2.45285
C	-5.55201	-0.000018	2.77978
H	-5.80501	0.000462	1.7213
H	-6.47261	0.000268	3.36804
C	5.55221	-0.00244	2.7795
H	6.47287	-0.00295	3.36766
H	5.8051	-0.00204	1.72098
C	-1.40938	4.10311	2.45528
C	3.89916	2.85449	4.23134
H	3.557	3.56127	4.97219
C	-2.92395	-4.17235	2.32207
H	-3.31074	-5.0701	2.81254
H	-3.21441	-4.23862	1.26868
C	-4.63295	1.72624	4.36057
H	-5.06827	1.27249	5.23752
C	4.63497	1.72455	4.36055
H	5.07039	1.27059	5.23734
C	0.69683	-5.27308	2.74462
H	1.22676	-6.19559	2.96758
C	-3.8992	-2.8582	4.2294
H	-3.55717	-3.56562	4.9697
C	-2.92147	4.17108	2.32453
H	-3.21204	4.23806	1.27121
H	-3.30798	5.06859	2.81562

C	-0.69935	-5.27287	2.74479
H	-1.2295	-6.19522	2.96787
C	2.92165	-4.17317	2.32144
H	3.21192	-4.23926	1.26798
H	3.30827	-5.07111	2.81165
C	2.9242	4.17033	2.32503
H	3.31089	5.06758	2.81645
H	3.21495	4.23759	1.27178
C	-4.63511	-1.72842	4.35945
H	-5.07066	-1.27523	5.23658
C	4.63386	-1.73017	4.35907
H	5.06956	-1.27723	5.23625
C	-0.69787	-2.9262	2.19868
H	-1.23099	-2.00099	1.99569
C	3.89723	-2.85947	4.22892
H	3.55459	-3.56665	4.96917
C	0.69589	-2.9264	2.19852
H	1.22925	-2.00136	1.99538
C	-3.89641	2.85569	4.23116
H	-3.55349	3.56219	4.97191
C	-0.69566	2.9246	2.1996
H	-1.22898	1.99981	1.99521
C	0.69809	2.92443	2.19973
H	1.23124	1.99951	1.99544
C	0.69956	5.27034	2.74904
H	1.22968	6.19243	2.97331
C	-0.69663	5.27051	2.74892
H	-1.22655	6.19273	2.9731

Table S5. Cartesians coordinates of calculated $[Ag_4(\mathbf{1})_2]^{4+}$ at the B3LYP/LANL2DZ (for Ag element) and B3LYP/6-31G* (for other atoms) level of theory.

Atom	X	Y	Z
C	0	0	0.28057
N	0	0	1.44084
C	0	0	-1.18097
H	0	1.02651	-1.56115
H	0.88898	-0.51326	-1.56115
H	-0.88898	-0.51326	-1.56115

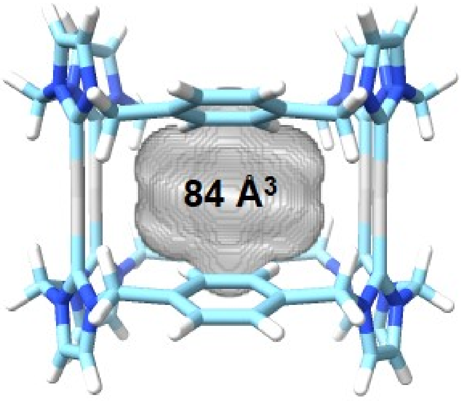

Figure S15. The corresponding calculated cavity volume of $Ag_4(\mathbf{1})_2(PF_6)_4$. The calculated cavity volume using the VOIDOO program (1.25 Å probe).[10] Color code: Ag, grey; N, blue, C, light blue, H, white. Counterions are omitted for clarity.

S7. References

1. Z. Fu, J. Lin, L. Wang, C. Li, W. Yan, T. Wu, *Cryst. Growth. Des.* **2016**, *16*, 2322–2327.
2. G. M. Sheldrick, SADABS, Program for Absorption Correction of Area Detector Frames, BRUKER AXS Inc, Madison, WI.
3. O. V. Dolomanov, L. J. Bourhis, R. J. Gildea, J. A. K. Howard, H. Puschmann, *J. Appl. Cryst.* **2009**, *42*, 339–341.
4. L. J. Bourhis, O.V. Dolomanov, R. J. Gildea, J. A. K. Howard, H. Puschmann, *Acta Cryst.* **2015**, *A71*, 59–75.
5. G. M. Sheldrick, *Acta Cryst.* **2015**, *C71*, 3–8.
6. Van der Sluis P V, Spek A L. Acta Crystallographica Section A: Foundations of Crystallography, **1990**, *46*, 194–201.

7. Frisch, M. J.; Trucks, G. W.; Schlegel, H. B.; Scuseria, G. E.; Robb, M. A.; Cheeseman, J. R.; Scalmani, G.; Barone, V.; Mennucci, B.; Petersson, G. A.; Nakatsuji, H.; Caricato, M.; Li, X.; Hratchian, H. P.; Izmaylov, A. F.; Bloino, J.; Zheng, G.; Sonnenberg, J. L.; Hada, M.; Ehara, M.; Toyota, K.; Fukuda, R.; Hasegawa, J.; Ishida, M.; Nakajima, T.; Honda, Y.; Kitao, O.; Nakai, H.; Vreven, T.; Montgomery, J. J. A.; Peralta, J. E.; Ogliaro, F.; Bearpark, M.; Heyd, J. J.; Brothers, E.; Kudin, K. N.; Staroverov, V. N.; Kobayashi, R.; Normand, J.; Raghavachari, K.; Rendell, A.; Burant, J. C.; Iyengar, S. S.; Tomasi, J.; Cossi, M.; Rega, N.; Millam, J. M.; Klene, M.; Knox, J. E.; Cross, J. B.; Bakken, V.; Adamo, C.; Jaramillo, J.; Gomperts, R.; Stratmann, R. E.; Yazyev, O.; Austin, A. J.; Cammi, R.; Pomelli, C.; Ochterski, J. W.; Martin, R. L.; Morokuma, K.; Zakrzewski, V. G.; Voth, G. A.; Salvador, P.; Dannenberg, J. J.; Dapprich, S.; Daniels, A. D.; Farkas, O.; Foresman, J. B.; Ortiz, J. V.; Cioslowski, J.; Fox, D. J.; Gaussian, Inc., Revision E.01. Wallingford CT, **2013**.
8. Hohenberg, P.; Kohn, W. Inhomogeneous Electron Gas. *Phys. Rev. B.* **1964**, *136*, B864−B871.
9. Kohn, W.; Sham, L. Self-Consistent Equations Including Exchange and Correlation Effects. *J. Phys. Rev.* **1965**, *140*, A1133−A1138.
10. G. J. Kleywegt, T. A. Jones, Acta Cryst. **1994**, *D50*, 178−185.
